# Disruption and recovery of river planktonic community during and after the COVID-19 outbreak in Wuhan, China

**DOI:** 10.1038/s43705-022-00168-7

**Published:** 2022-09-19

**Authors:** Junwen Chen, Weixiao Qi, Donglin Wang, Qiaojuan Wang, Hui Lin, Guannan Mao, Jinsong Liang, Xue Ning, Yaohui Bai, Huijuan Liu, Jiuhui Qu

**Affiliations:** 1 Center for Water and Ecology, Tsinghua University, Beijing, 100084 China; 2 Key Laboratory of Drinking Water Science and Technology, Research Center for Eco-Environmental Sciences, Chinese Academy of Sciences, Beijing, 100085 China; 3 School of Civil and Environmental Engineering, Harbin Institute of Technology, Shenzhen, 518055 China; 4 MaREI Centre, Environmental Research Institute, School of Engineering, University College Cork, Cork, T23XE10 Ireland

**Keywords:** Environmental sciences, Water microbiology

## Abstract

During the COVID-19 outbreak in Wuhan, large amounts of anti-coronavirus chemicals, such as antiviral drugs and disinfectants were discharged into the surrounding aquatic ecosystem, causing potential ecological damage. Here, we investigated plankton in the Wuhan reaches of the Yangtze River, before, during, and after COVID-19, with the river reaches of three adjacent cities sampled for comparison. During the COVID-19, planktonic microbial density declined significantly. Correspondingly, the eukaryotic and prokaryotic community compositions and functions shifted markedly, with increasing abundance of chlorine-resistant organisms. Abundance of antibiotic resistance genes, virulence factor genes, and bacteria containing both genes increased by 2.3-, 2.7-, and 7.9-fold, respectively, compared to other periods. After COVID-19, all measured plankton community compositional and functional traits recovered in the Yangtze River.

## Introduction

In December 2019, the city of Wuhan in China experienced an outbreak of a novel coronavirus disease (COVID-19), which shows greater lethality and transmission than the flu [1]. During the pandemic, considerable quantities of antivirals and antibiotics were used to treat the novel virus and associated bacterial infections in patients [2, 3]. Furthermore, to prevent environmental transmission of COVID-19, at least 2 000 tons of disinfectants (e.g., hypochlorous acid and peracetic acid) were used both indoors and outdoors in Wuhan to inactivate the virus [4] (Supplementary Text 1). Consequently, residual anti-coronavirus chemicals (drugs and disinfectants) were discharged into the surrounding streams, rivers, and lakes from sewage treatment plants and surface runoff, threatening aquatic communities. Previous laboratory experiments have demonstrated that exposure to chlorine disinfectants or antibiotics may change microbial community composition, increase antimicrobial resistance through rapid mutation and gene transfer [5], and affect higher trophic level organisms, e.g., shrimp, in receiving aquatic ecosystems [6]. However, whether these outcomes occur in the field and disappear in the absence of stress remains unclear.

**Fig. 1:**
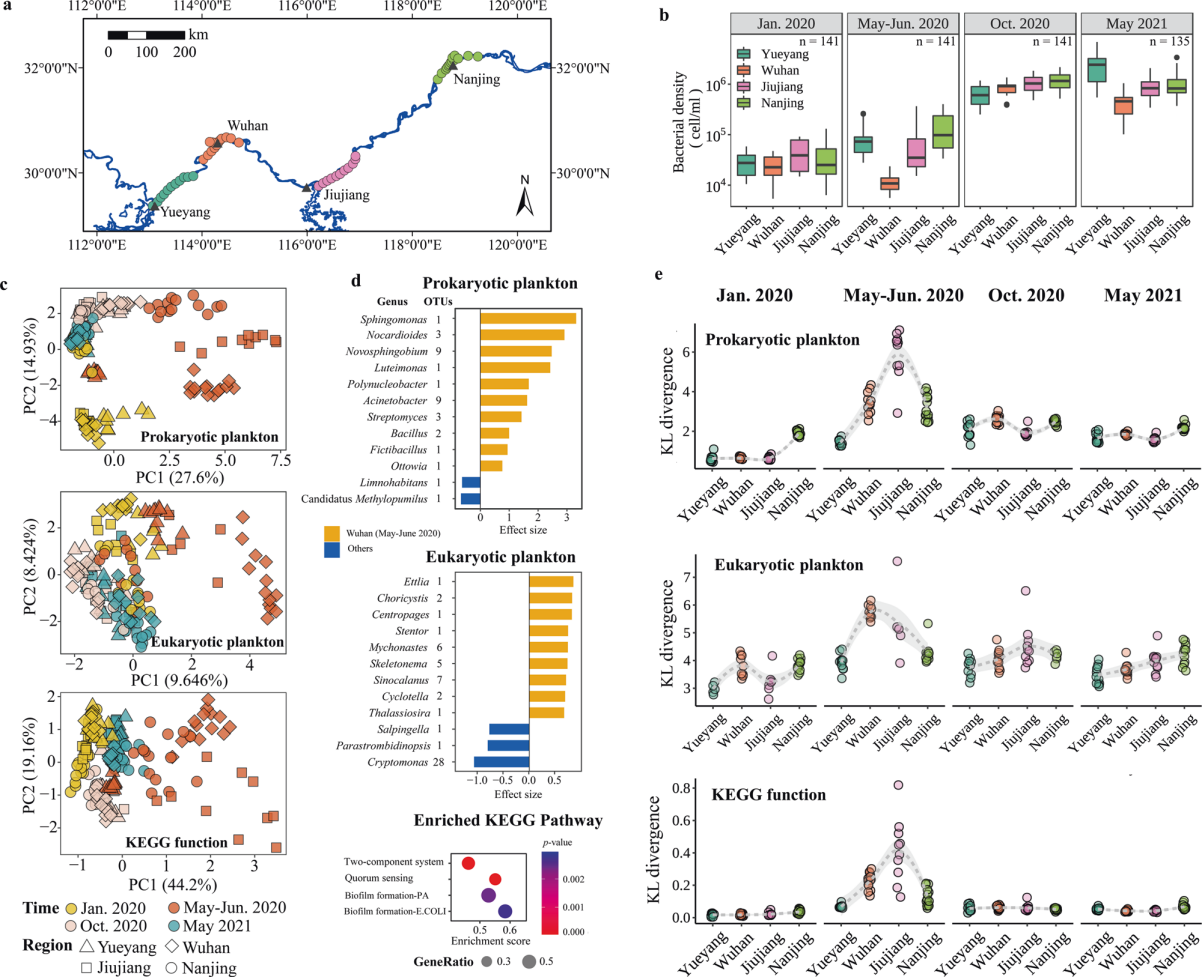
Disruption and recovery of Yangtze River plankton during and after COVID-19 outbreak in Wuhan, China. **a** Surface water sampling was conducted along the Yangtze River, including the Wuhan, upstream, and downstream reaches (~960 km), before (January 2020), during (May–June 2020), and after (October 2020 and May 2021) COVID-19 in Wuhan. **b** Total bacterial cell density of water samples (*n* = 558, three replicates per sample) across four reaches before, during, and after COVID-19, was measured by flow cytometry. **c** PCA analysis was performed, showing clear separation (ANOSIM, Adonis: *p* < 0.05) between Wuhan and Jiujiang samples in May–June 2020 and other samples in both community composition (prokaryotic and eukaryotic plankton) and function. **d** Marked differences were observed between Wuhan-May-2020 samples and other samples in prokaryotic and eukaryotic composition and KEGG pathways. Accordingly, compared to other samples, significant differential OTUs in Wuha-May-2020 (Wilcoxon test, Benjamini–Hochberg corrected *p* < 0.01) and highly abundant KEGG pathways (adjusted *p* < 0.05, gene set enrichment analysis (GSEA) with clusterProfiler R package) were displayed. More details are provided in Supplementary Tables 8–10. **e** To assess plankton recovery, KL divergence (relative entropy comparing species/function distribution) compared to reference sample (upstream Yueyang in January 2020) was calculated. Smooth curves (loess, span = 1) were obtained to show patterns more visually. Pair contrasts between different groups showed abrupt disturbance in May–June 2020 and recovery in October 2020 and May 2021.

To address these issues, we completed sampling campaigns in the Wuhan reaches of the Yangtze River, which is the largest river in China and also one of the main reservoirs receiving residual anti-coronavirus chemicals from wastewater treatment plants and runoff in Wuhan, as well as the upstream (Yueyang) and downstream reaches (Jiujiang and Nanjing) (minor COVID-19 disturbance) for comparison (Fig. 1a). In total, 186 surface water samples (~0.5 m in depth) were collected along the Yangtze River from the four reaches (across 960 km) before (January 2020), during (May to June 2020), and after (October 2020 and May 2021) COVID-19 outbreak (intensive use of anti-coronavirus chemicals) in Wuhan (Supplementary Table 1). We analyzed the chemical properties, total cell density, and metagenomic sequences of the water samples.

## Results and discussion

The residual chlorine in the Wuhan-Yangtze River reaches in May 2020 was 0.025 ± 0.01 mg/L (Supplementary Table 2), exceeding the chlorine level (0.019 mg/L) that may cause acute toxicity in freshwater organisms [7]. The flow cytometry results (Fig. 1b and Supplementary Table 3) showed that microbial cell density in the Wuhan samples was 1.16 × 10^4^ ± 0.42 × 10^4^ ml^−1^ during the COVID-19 outbreak, which was lowest among all the groups (Wilcoxon test, *p* < 0.01). Furthermore, by establishing the linear mixed model, we found that after eliminating the effect of other environmental variables, including nutrients, precipitation and temperature, the impact of COVID-19 on bacterial cell density in May–June 2020 was still significant (*t*-test, *p* < 0.05, Supplementary Text 2). The above results suggested that discharge of anti-coronavirus chemicals into the Yangtze River decreased plankton microbial cells.

We used principal components analysis (PCA) to discern the spatiotemporal changes in plankton community composition and function in the different reaches. The PCA and analysis of similarity (ANOSIM) results were consistent for prokaryotic and microeukaryotic plankton and community function (Fig. 1c and Supplementary Tables 4–6). A significant segregation between the Wuhan-May-2020 samples and other samples (ANOSIM, *p* < 0.001) was observed. By fitting the composition data with a neutral model, we demonstrated that the differences in microbial composition in May–June 2020 were dominated by environmental selection, not by stochastic process (Supplementary Text 3 and Supplementary Fig. 1). In addition, the alpha diversity (operational taxonomical unit (OTU) richness and Shannon diversity) of prokaryotic and microeukaryotic plankton in Wuhan decreased during COVID-19 (Supplementary Fig. 2 and Supplementary Table 7). Overall, the above results indicated that both plankton community composition and function in the Wuhan-Yangtze River section were significantly affected during the COVID-19 outbreak.

Accordingly, differences in microeukaryotic and prokaryotic species and functional profiles in the Wuhan-May-2020 samples and other samples were examined. We filtered 31 highly abundant prokaryotic OTUs in Wuhan-May-2020 (Fig. 1d and Supplementary Table 8), which were attributed to 10 genera including *Acinetobacter*, *Novosphingobium*, *Nocardioides*, *Streptomyces* and *Bacillus*. Among them, *Acinetobacter*, *Novosphingobium*, and *Bacillus* were often referred as chlorine resistant [8–10] and some strains exhibited the potential to degrade chlorinated compounds [11, 12]. Similarly, we filtered 26 highly abundant microeukaryotic OTUs in Wuhan-May-2020 (Fig. 1d and Supplementary Table 9), which belonged to nine genera including *Sinocalanus*, *Cyclotella*, *Skeletonema*, *Mychonastes*, and *Choricystis*. Among them, *Cyclotella* and *Choricystis* were documented as chlorine resistant or living in chlorinated water [13, 14], while *Mychonastes* was able to remove cephalosporin antibiotics in wastewater [15]. Moreover, we also found the relative abundance of metagenome-assembled genomes (MAGs) carrying genes associated with chlorine resistance increased significantly (*t*-test, *p* < 0.001) in Wuhan during the COVID-19 (Supplementary Text 4 and Supplementary Fig. 3). These results indicated that the abundance of chlorine-resistant organisms in the Wuhan reaches increased during the COVID-19. Kyoto Encyclopedia of Genes and Genomes (KEGG) pathway functional analysis identified 17 significantly enriched pathways, including eight up-regulated and nine down-regulated pathways (Supplementary Table 10). The up-regulated pathways included biosynthesis of siderophore and lipoarabinomannan, quorum sensing, biofilm formation, and two-component signal transduction systems (Fig. 1d), consistent with previous observations and genetic findings that chlorine disinfectants enhance quorum sensing and biofilm formation [16, 17].

To assess the temporal variation trend in plankton community composition and function, we calculated Kullback–Leibler (KL) divergence (relative entropy comparing species or functional composition) [18] from a reference sample (Yueyang-6-January 2020). Remarkably, although KL divergences in community species and functional composition showed a sharp increase in Wuhan and Jiujiang in May–June 2020 (Fig. 1e, *p* < 0.001, and Supplementary Tables 11–13), they all returned to the pre-COVID-19 levels from October 2020 to May 2021, suggesting recovery of the plankton community after COVID-19 disturbance.

**Fig. 2:**
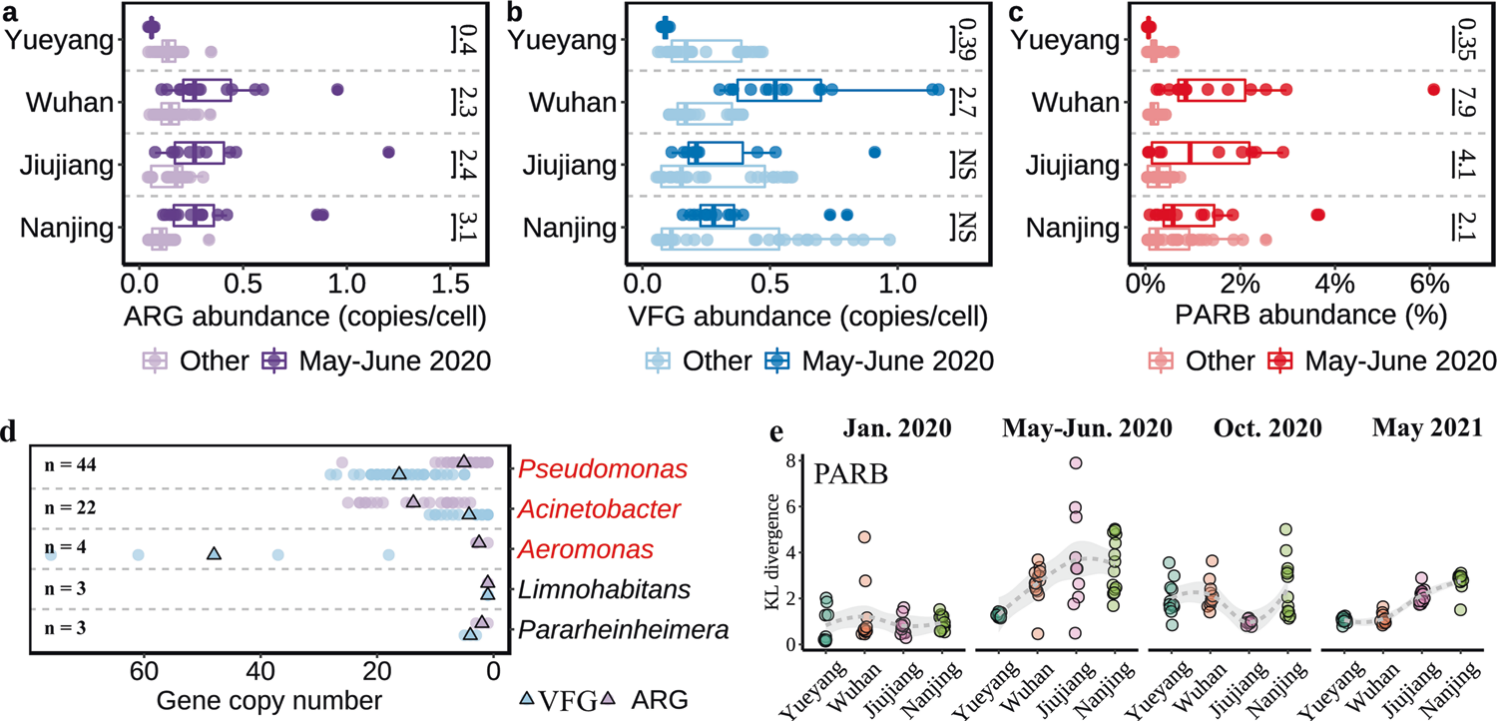
Response of antimicrobial resistance and virulence factors to COVID-19 outbreak in Wuhan. To address potential health-related microbial risk during and after COVID-19, abundances (copy number/cell) of antibiotic resistance genes (ARGs) (**a**), virulence factor genes (VFGs) (**b**), and pathogenetic and antimicrobial resistant bacteria (PARB, containing both ARG and VFG) (**c**) were compared between May–June 2020 and January 2020, October 2020, and May 2021 (NS: non-significant; numbers mean significant fold changes between groups). Among the 89 identified PARB, 85.4% were attributed to the top five most abundant genera (**d**); ARG or VFG copies were mostly enriched in *Pseudomonas*, *Acinetobacter*, and *Aeromonas* (in red), triangle represent average gene copy number. However, KL divergence analysis (**e**) showed that PARB composition gradually returned to their original January 2020 state.

To verify that exposure to chlorine and antiviral drugs may increase antimicrobial resistance and microbial virulence factors in the Yangtze River, we explored the distribution patterns of antibiotic resistance genes (ARGs), virulence factor genes (VFGs), and bacterial genomes containing both ARGs and VFGs, defined as pathogenetic and antimicrobial resistant bacteria (PARB) [19]. Results showed that ARG abundance (copy number/cell) in Wuhan and the lower reaches of Jiujiang and Nanjing was significantly higher in May–June 2020 than in other periods (Fig. 2a, Wilcoxon test, *p* < 0.01), with increases in multidrug, beta-lactam, and macrolide-resistant gene types (Supplementary Fig. 4). Notably, almost all bacteria (99.2%) containing ARGs had mobile genetic elements (Supplementary Table 14 and Supplementary Fig. 5), suggesting frequent horizontal gene transfer [20]. Consistently, VFG abundance (copy number/cell) in Wuhan was also significantly higher in May–June 2020 than that in other periods (Fig. 2b, Wilcoxon test, *p* < 0.001), which was mainly induced by the increases in the abundance of bacteria containing VFGs (Supplementary Table 15) and motility VFG type (Supplementary Fig. 6). Furthermore, the relative abundance of PARB in Wuhan-May-2020 was 1.56% (Supplementary Table 15), which was 7.9 times higher than the abundance in Wuhan during other periods and was the highest among all groups (Fig. 2c). Among the 89 identified PARB, 78.7% were attributed to *Pseudomonas*, *Acinetobacter*, and *Aeromonas* (Fig. 2d), some strains of which were known as human opportunistic pathogens, such as *Acinetobacter baumannii* and *Pseudomonas aeruginosa*. Although PARB cell density in May–June 2020 was low (Supplementary Table 16), the high ARG and VFG abundances in PARB (Fig. 2d) highlighted the potential secondary health risks of intensively used disinfectants and drugs. Remarkably, both PARB abundance (Supplementary Table 15) and composition in Wuhan returned to the original level in October 2020 and May 2021 (Fig. 2e and Supplementary Table 17).

In summary, we demonstrated that the discharge of residual disinfectants and drugs during the COVID-19 outbreak disturbed the aquatic community in Wuhan, resulting in decreased microbial cell density and alpha diversity, increased abundance of ARGs, VFGs, and PARB, and compositional and functional changes, with higher abundance of chlorine-resistant species and genes. Notably, after the outbreak, all measured plankton community compositional and functional traits recovered in the Yangtze River. This was likely due to the termination of intense disinfection and drug use and the high flow rate (Supplementary Table 18) of the river, which diluted and transported the residual chemicals downstream.

## Materials and methods

### Study areas and sampling campaigns

The Yangtze River was the main reservoir receiving antiviral and disinfectant discharge from wastewater treatment plants and runoff in Wuhan during the COVID-19 outbreak (defined as intense use of anti-coronavirus drugs and disinfectants which originated in late January 2020 and ended in April 2020 and July 2020, respectively). The Yangtze River is the largest river in China, originating from the Tibetan Plateau and flowing into the East China Sea at Shanghai. We collected surface water samples (0–50 cm in depth) from four reaches of the Yangtze River (i.e., Yueyang, Wuhan, Jiujiang and Nanjing, Fig. 1a) before (1–15 January 2020), during (1–3 May to 17–30 June 2020), and after (17–24 October 2020 and 17–28 May 2021) the COVID-19 outbreak in Wuhan (Supplementary Table 1). In total, 190 water samples were obtained. Water temperature, conductivity, oxidation-reduction potential (ORP), total dissolved solids (TDS), and pH were measured in situ using a multi-parameter water quality sonde (Myron Co., California, USA). Dissolved oxygen (DO) was measured in situ using a portable dissolved oxygen meter (HACH Co., Colorado, USA). The residual chlorine concentration was measured in situ using a pocket colorimeter DR300 (HACH co., USA). Approximately 100 mL of water was collected for chemical analysis of total nitrogen, dissolved organic carbon, and total phosphorus (Supplementary Table 2).

Surface water (1 mL) was loaded into centrifuge tubes for microbial cell quantification using flow cytometry. Approximately 900–1800 mL of surface water was filtered using 0.45-μm filter membranes (PCTE, GVS, Bologna, Italy) in the field with a pump, followed by the addition of LifeGuard™ preservation solution (MoBio Laboratories Inc., Carlsbad, CA, USA). All samples were transported to the laboratory on ice.

### Flow cytometry

Flow cytometry and fluorescent staining were carried out based on previously described methods [21, 22]. For total cell concentration, 500 μL of sample (pre-warmed for 3 min at 37 °C) was stained with 5 μL of SYBR Green I (SGI) (100 × diluted; Invitrogen, California, USA), then mixed and incubated in the dark for 10 min at 37 °C [23]. Flow cytometry analysis was performed on an ACEA NovoCyte^TM^ (ACEA, California, USA) equipped with a 20-mW laser emitting at a fixed wavelength of 488 nm. Bacterial signals were selected using electronic gating on density plots of green (FL1; 533 nm) and red fluorescence (FL3; > 670 nm). Measurements were performed at a pre-set flow rate of 66 µL/min. The flow cytometer was regularly calibrated with calibration beads. All data were processed using ACEA NovoExpress^TM^ software (v1.9.1).

### DNA extraction and metagenomic sequencing

Total DNA was extracted from the membrane filters using a PowerWater^®^ DNA Isolation Kit (Qiagen, Hilden, Germany) following the manufacturer’s instructions. The extracted DNA samples (*n* = 190) were sent to the Beijing Genomics Institute (China) for 150-bp paired-end shotgun metagenomic sequencing using the Illumina HiSeq 2500 sequencing platform. After DNA quality check, the amount of DNA in four samples (JJ6_6_2020, YY11_1_20, WH5_1_20, and NJ11_10_20) was insufficient for library construction. Detailed sequencing information is listed in Supplementary Table 19. In total, 2105 Gb of sequencing data from 186 samples were obtained. All clean reads were deposited in the China National Center for Bioinformation database (https://www.cncb.ac.cn) under accession number CRA006054.

### Characterization of prokaryotic and eukaryotic plankton community composition

To determine the plankton community composition, we extracted 16 S rRNA and 18 S rRNA gene fragments from the filtered clean reads using Metaxa2 [24]. The recovered 16 S fragments were clustered using USEARCH (v10.0.240) [25] and taxonomically classified was performed against the SILVA release 138 [26] using best-match hits in the Ribosomal Database Project (RDP) at a similarity of ≥97% in QIIME1 (v1.9.1) [27]. Recovered 18 S fragments were clustered using USEARCH (v10.0.240) and taxonomically classified against the Protist Ribosomal Reference database (PR^2^, v4.13.0) using *uclust* at a similarity of ≥97% in QIIME1 (v1.9.1). Alpha and beta diversity indices were calculated in QIIME1. After centered log-ratio transformation of the compositional data, PCA analysis was performed using the *phyloseq* package (v1.34.0) in R.

The ANOVA-like differential expression (ALDEx2) [28] was adopted to identify differentially abundant species between Wuhan-May-2020 samples and others (Jiujiang-June-2020 samples were excluded due to the potential effect of the water bodies in Wuhan river reaches on downstream water bodies). Species with average abundance >0.1% and Benjamini–Hochberg corrected *p* < 0.01 were filtered. The 31 highly abundant and 2 low abundant bacteria, as well as the 26 highly and 30 low abundant microeukaryotic species, were identified.

### Characterization of bacterioplankton community function

The 186 samples were divided into 16 groups (Supplementary Table 20) based on their reaches and sampling time and co-assembled using MEGAHIT [29], with each group containing ~100 Gb of raw data. The selected assembled contigs (≥500 bp in length) were used to predict open reading frames (ORFs) with Prodigal (v2.6.3) [30], and all ORFs were merged and clustered with CD-EST-HIT (v4.7; -c 0.95 -aS 0.9 -g 1) [31] to generate a non-redundant gene library. BBMap (v38.43) [32] was used to map each metagenomic read to the non-redundant gene library and to normalize read counts to per kilobase per million mapped reads (RPKM). Combined with functional annotation of the non-redundant gene library, which was performed using eggNOG-mapper (v1.0.3) [33], the abundance of KEGG orthologs in each sample was acquired.

PCA was used to reveal functional differences in patterns among samples from different river reaches and collection times. According to the results, we classified the functional matrix into two groups: Wuhan-May-2020 and others (excluding Jiujiang-June-2020). We identified differentially expressed genes (DEGs) between the two groups using DESeq in R. DESeq log2 fold-change was determined with the shrinkage estimator apeglm. Genes were considered differentially abundant with an adjusted *p* < 0.05. KEGG pathway enrichment analysis was based on the abundance of corresponding KEGG orthologs using the R package clusterProfiler (v3.18.1).

### Classification and quantification of ARGs and VFGs

To identify and quantify ARGs in samples, clean reads of each sample were processed using the ARGs-OAP v2.2 pipeline [34]. The ARGs sequences were identified against SARG v2.2, a database that sorts ARG-like sequences in a hierarchical structure (type-subtype-reference sequence). The abundance of ARGs was normalized against the ARG reference sequence length and the cell numbers with a unit of “ARGs copies per cell”.

To identify and quantify VFGs in the samples, clean reads of each sample were mapped to the core VFG sequences from the virulence factor database (VFDB) [35] (http://www.mgc.ac.cn/VFs/download.htm), with an aligner in BBMap (v38.73) using slow mode (high sensitivity). The core VFG sequences were downloaded on 13 April 2021 and were classified into type and subtype referring to our previous research [19]. The copy number of each virulence factor gene type and subtype was calculated by adding the coverage value of all VFG sequences belonging to a specific subtype or type. The abundance of VFGs at the type and subtype level was normalized based on the cell number (estimated by the ARGs-OAP v2.2 pipeline) in each sample with a unit of “VFGs copies per cell”.

### Genome-centric analysis to identify bacterial strains carrying ARGs, VFGs, and both

Genomic binning was conducted according to the MetaWRAP pipeline [36] to recover metagenomic assembled genomes (MAGs) (completeness > 50%; contamination < 10%). The obtained MAGs were de-replicated using dRep [37] to generate a non-redundant MAG dataset containing 7376 MAGs. To quantify the relative abundance of the MAGs in each sample, BBMap (v38.43) was used with custom script (MAG mapping ratio supported in Supplementary Table 21). The MAGs were taxonomically classified using GTDB-Tk (v0.2.2) [38].

We further identified MAGs containing ARGs and VFGs [19]. First, the open reading frames (ORFs) in all contigs of each MAG were predicted using Prodigal (v2.6.3). Then, to identify potential ARG ORFs, the ORFs were aligned against the SARG database in ARG-OAP v2 [34] using BLASTP [39]. An ORF was regarded as a potential ARG sequence when it shared at least 80% similarity over more than 70% of the length of the top hit in the database. Potential VFG ORFs were also identified using identical criterion for BLASTP alignment against protein sequences down from the VFDB database [35].

MAGs containing both ARGs and VFGs were regarded as potential PARB. In total, we identified 653 and 190 MAGs containing ARGs and VFGs, respectively, with 89 MAGs containing both (Supplementary Tables 22–24).

### Genome-centric analysis to identify bacterial strains carrying both ARGs and MGEs

The ORFs from each MAGs were subjected to a BLASTP search against the plasmid protein database ACLAME (http://aclame.ulb.ac.be) with an E value threshold of e-10, 80% sequence similarity cutoff, and 70% coverage [40]. The MAGs contained both the ARGs and mobile gene elements (MGEs) were considered as microbes with potential ARG transferability.

### Statistical analysis

All analyses were performed in *R* (v4.0.2) [41]. Wilcoxon tests were performed for pair comparisons of microbial cell densities among reaches and collection times, as well as comparisons of abundances of ARG, VFG, PARB, and prokaryotic and eukaryotic species between Wuhan-May-2020 and 14 other groups (excluding Jiujiang-June-2020). Analysis of similarity (ANOSIM), permutational multivariate analysis of variance using distance matrices (Adonis), and multi response permutation procedure (MRPP) were performed to test differences in prokaryotic and eukaryotic composition and community function differences among reaches and times using the vegan package (v 2.5-7).

KL divergences were calculated to represent divergences of species or functional probability distributions from different samples using the philentropy package (v0.5.0) [42]. To explore the influence of river reaches and sample times on KL divergence relative to the reference state (Yueyang-January-2020), KL divergences were further fitted with the generalized least squares model, implemented in the nlme package (v3.1-153). Besides, the pair comparisons of KL divergences of each group were conducted to verify the abrupt disturbance in May–June 2020 and recovery in October 2020 and May 2021 using the emmeans package (v1.7.2).

## Supplementary information

Supplementary Information

Supplementary Table 14,22

## Data Availability

All the sequence data were deposited in the China National Center for Bioinformation database (https://www.cncb.ac.cn) under accession number CRA006054. The codes during the current study are available from the corresponding author on reasonable request.
