# Supplementary material for: Disruption and recovery of river planktonic community during and after the COVID-19 outbreak in Wuhan, China
Source: ISME Commun. 2022 Sep 19;2:84. doi: 10.1038/s43705-022-00168-7 (PMC9483884; doi:10.1038/s43705-022-00168-7)
Supplement: Supplementary file 1 — Supplementary Information [file 43705_2022_168_MOESM1_ESM.docx]

**Supplementary information**

**Disruption and recovery of river planktonic community during and after the COVID-19 outbreak in Wuhan, China**

**Supplementary text**

**Information on antiviral chemical data in Wuhan (Supplementary Text 1).**

According to a survey (unpublished) conducted by the Chinese Academy of Environmental Sciences from February to March 2020, the type of the disinfectants used in Wuhan at that time was mainly chlorine-based disinfectant with sodium hypochlorite in sewage treatment and chlorine dioxide in medical wastewater. During this period, 26 sewage treatment plants in Wuhan continuously used sodium hypochlorite for 24h, with a cumulative total of 1777.36 tons of wastewater and 33.69 tons of sludge. For the antiviral drugs, dozens of antiviral drugs such as chloroquine phosphate, ribavirin and lamivudine have been used since February 2020. Antibiotics were heavily used in the early stages of the COVID-19, and mainly used to deal with lung bacterial infections for severe COVID-19 patients in the middle and late stages.

**Mixed linear model (Supplementary Text 2)**

To better understand the effect of COVID-19 on the bacterial cell density in Wuhan reaches during the COVID-19, we adopted a mixed linear model (formula 1) in our analysis. We assumed that without COVID-19 in Wuhan, the predicted bacteria cell density of the model should be close to the observed one (i.e., the difference is not significant).

$\text{Log}_{10}(\text{TCD})=\text{DO}+\text{Temp}+\text{TOC}+\text{TN}+\mathrm{Precipitation}+\text{Waterflow}+(1|\text{Time})+(1|\mathrm{Region})$ (1)

To achieve normality, we first performed Box-Cox transformation by taking the logarithm of bacterial cell density. Then we examined the collinearity of the continuous variables by correlation matrices. Next, we applied the “*lmer*” function in the R package lme4 (V 1.1-29) to obtain the estimated coefficients where bacterial density is the dependent variable; temperature, dissolved oxygen, total organic carbon, total nitrogen, total phosphorus, rainfall, and water flow are fixed effects, and sample time and city reaches are modeled as random effects.

The model showed good predictability as the predicted cell densities have no significant differences to the observed cell densities in almost all the groups (Supplementary Text 2 Figure). However, the predicted bacterial cell density in Wuhan May-June 2020 was significantly higher than the observed bacterial cell density after controlling for sample time, regions, and the environment variables (t-test, *p* < 0.05), while not significant (t-test, *p* = 0.26) in May 2021 (Supplementary Text 2 Figure). The above conclusion still hold even we trained the model without data from May-June 2020, and May 2021. The result was contradictory to the previous assumption, suggesting the un-observed variables, for example the residual disinfectants during COVID-19, might lead to decreased bacterial cell density in Wuhan May-June 2020.


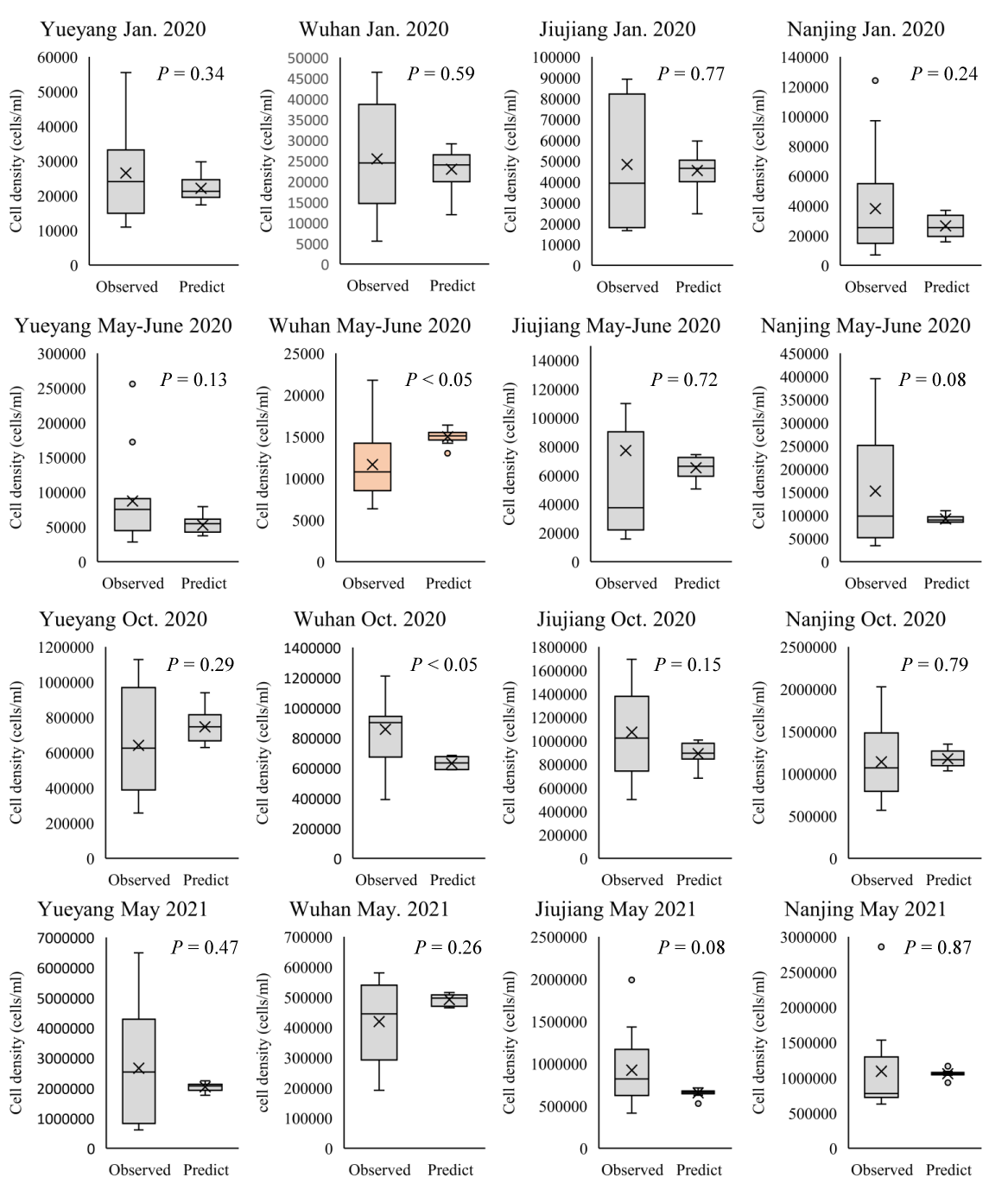


**Supplementary Text 2 Figure: The boxplot of predicted and observed bacterial density for each group. The subfigure with light orange is the group of Wuhan May-June 2020.**

**Community neutral model (Supplementary Text 3)**

To determine the importance of neutral process on community assembly, we used a neutral community model (NCM) to predict the relationship between OTUs detection frequency and their relative abundance in the metacommunity ^1^. In general, the taxa with high relative abundance tends to be widespread due the neutral dispersal process. The parameter Nm calculated from the data is an estimate of community dispersal with N representing the metacommunity size and m describing the migration rate. Parameter R^2^ represent the model fit to the observed data ^2^.

**Identification of chlorine resistant bacteria (Supplementary Text 4)**

Bacteria respond to chlorine toxicity differently than antibiotics. Bacteria can deal with specific antibiotic by acquiring corresponding resistant genes. However, chlorine was nonspecific and usually do harm to multiple cell target: such as cell walls, cell membranes and genetic materials. This leads to the fact that when bacteria under chlorine stress, the functional genes, including oxidative stress, cell wall repair, quorum sensing, and antioxidant organic matter synthetic (like glutathione) increased. So, to identify the potential chlorine resistant bacteria, we searched on the “web of science” with AB = (“chlorine” AND “gene” AND “micro*”) and found 161 published papers, after filtering the unrelated papers. We extracted functional genes which were observed overexpression or with high abundance when treated with chlorine. These genes were regarded as potential chlorine-resistant genes. The related sequences were manually curated from NCBI and deposited in https://github.com/culsome/Anti-chlorine-database. To identify the potential chlorine-resistant microbes, the nucleotide sequences of each MAG were subjected to a blastn search against the custom chlorine-resistant gene database (E value < e-10, identity > 80% and coverage > 70%).

**Supplementary Figures**


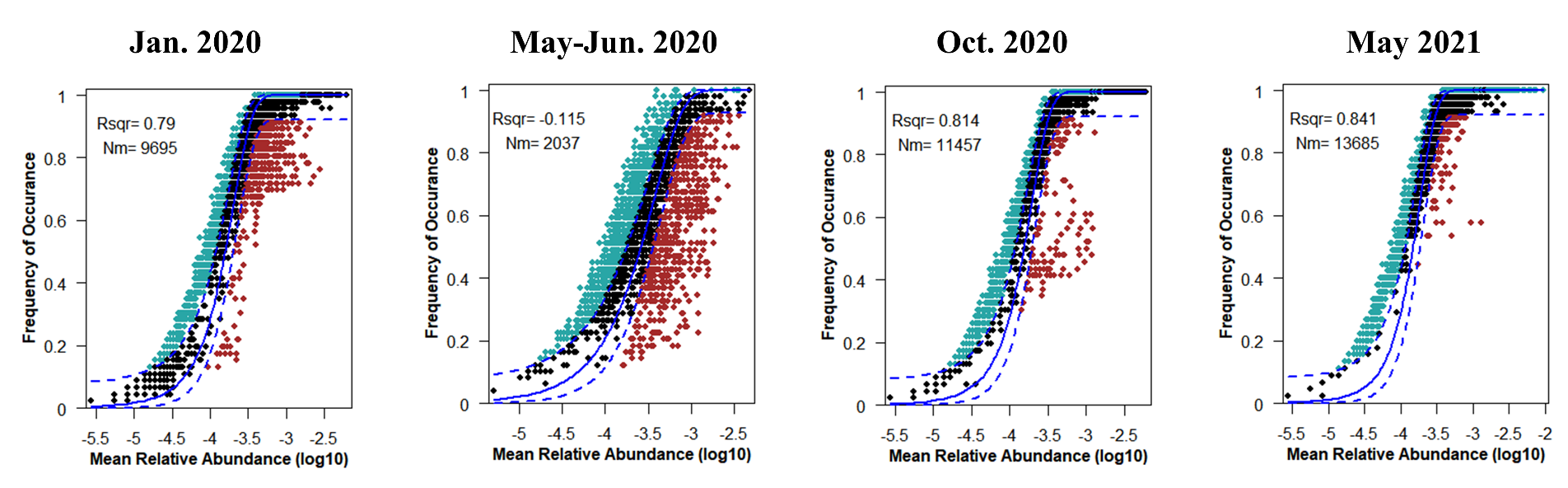


**Supplementary Figure 1.** **Fit of neutral community model (NCM) to prokaryotic community.** Predicted prokaryotic frequencies of occurrence in each period are shown. Solid blue lines indicate best-fit to NCM, dashed blue lines represent 95% confidence interval around best-fit prediction. OTUs lower or higher than dashed lines are shown in red and light blue, respectively, and indicate they occur less or more frequently than predicted NCM fit. Nm indicates metacommunity size (N) × immigration (M). R^2^ indicates fitness to model. Model was developed and modified according to previous research ^2,3^.


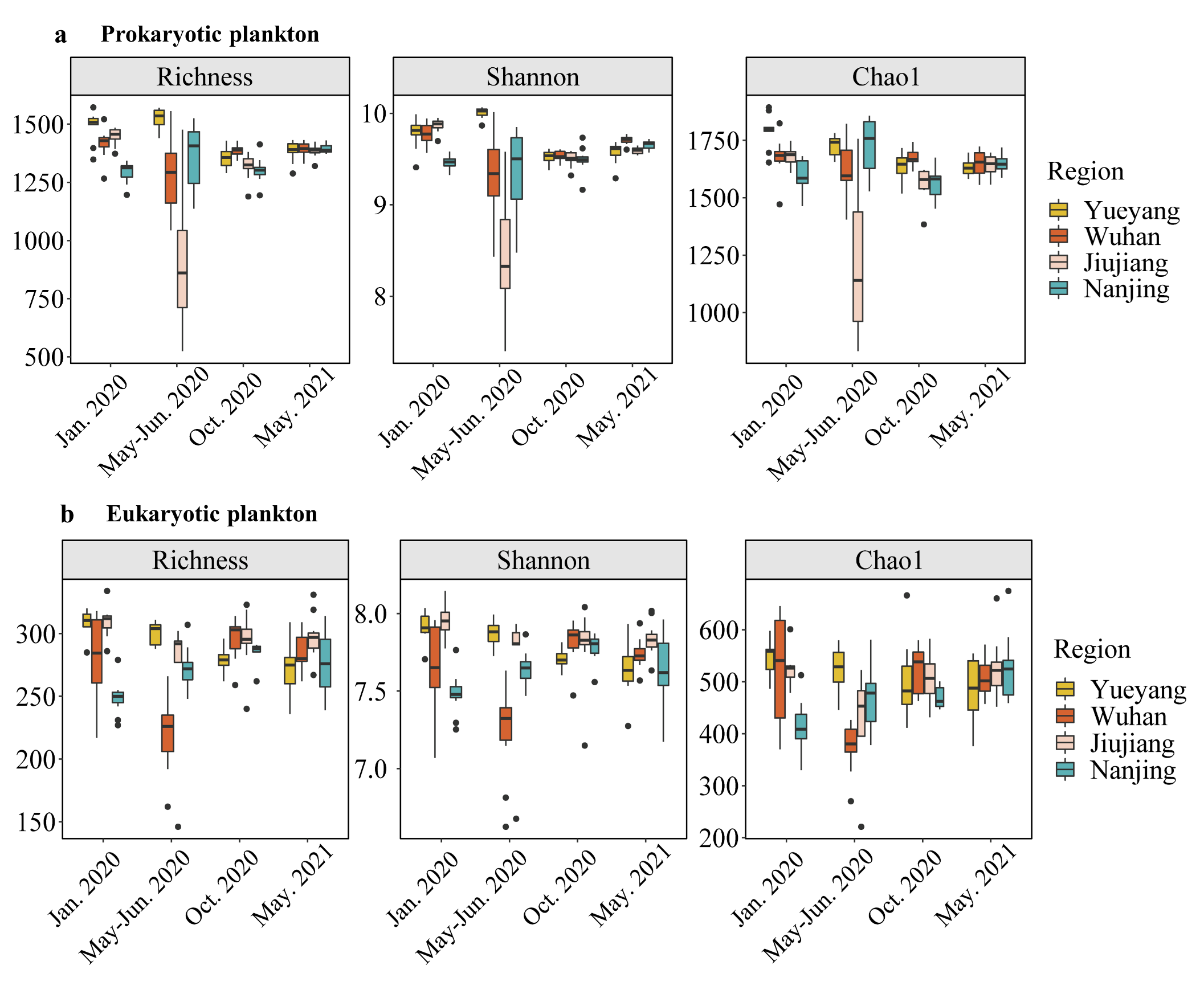


**Supplementary Figure 2. Alpha diversity (richness, Shannon, and Chao1) of prokaryotic and eukaryotic plankton at each period across four cities along the Yangtze River**. The alpha diversities were calculated according to the relative abundance of prokaryotic and eukaryotic OTUs in each sample. The base of Shannon index was natural logarithms.


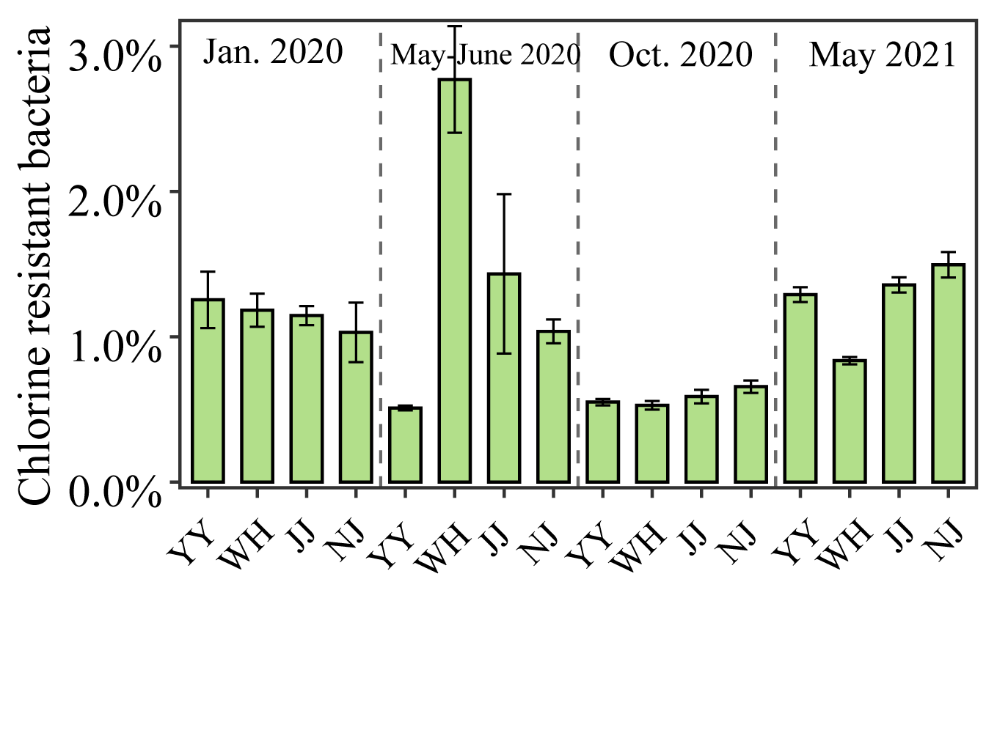


**Supplementary Figure 3. Relative abundance of chlorine resistant bacteria (based on genome level) among reaches and times.** Detail for identifying chlorine resistant bacteria, see Supplementary Text 4. Chlorine resistant bacteria abundance in Wuhan May-June 2020 was significantly higher than that in other groups (t-test, *p* < 0.001)


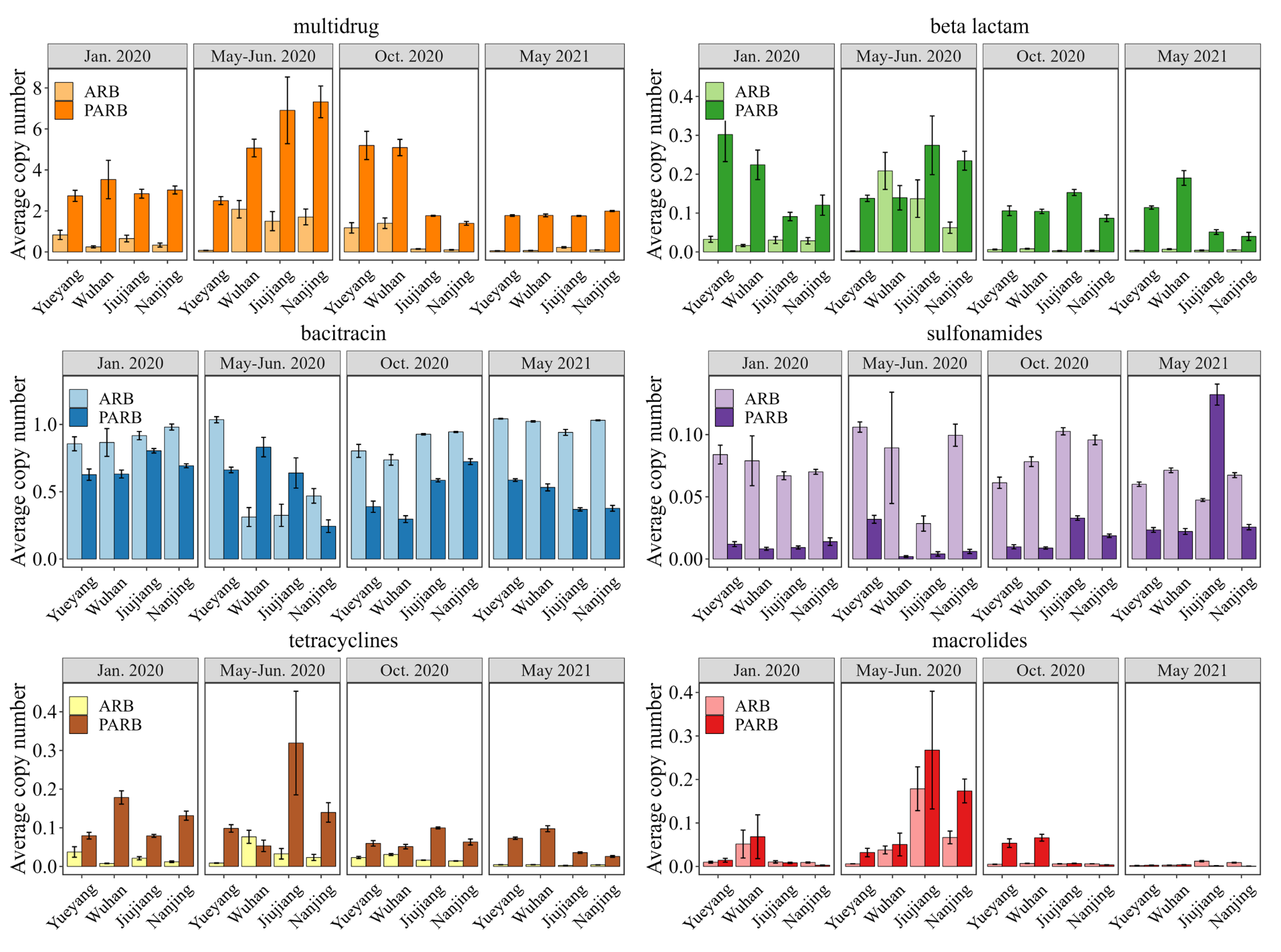


**Supplementary Figure 4. Abundance (copy number/cell) of top 6 abundant antibiotic resistance gene (ARG) types among reaches and times.** The antibiotic resistant bacteria (ARB) here is defined as bacteria containing ARG(s) without VFG(s), pathogenetic and antimicrobial resistant bacteria (PARB) is defined as bacterial containing both ARG(s) and VFG(s). The calculated formulas of copy number of ARB and PARB in specific ARG type were follows:

$\text{ARG}_{\text{ARBcopies}}=\frac{\sum_{i=1}^{m} ARB_{i}\times ARGtype_{i}}{\sum_{i=1}^{m} ARB_{i}}$, $\text{ARG}_{\text{PARBcopies}}=\frac{\sum_{i=1}^{k} PARB_{i}\times ARGtype_{i}}{\sum_{i=1}^{k} PARB_{i}}$, where *ARB_i_* is the relative abundance of ARB *i* in the sample, *ARGtype_i_* is number of specific ARG type (i.e., multidrug, beta-lactam) copies in ARB *i* and *m* is the number of ARB in the sample. P*ARB_i_* is the relative abundance of PARB *i* in the sample, *ARGtype_i_* is the number of specific ARG type copies in PARB *i* and *k* is the number of PARB in the sample. The multidrug, beta-lactam, and macrolides resistant gene copies of ARB and PARB of Wuhan reaches and the downstream Jinjiang and Nanjing were significantly higher in May-June 2020 than the other periods (Wilcoxon test, *p* < 0.05).


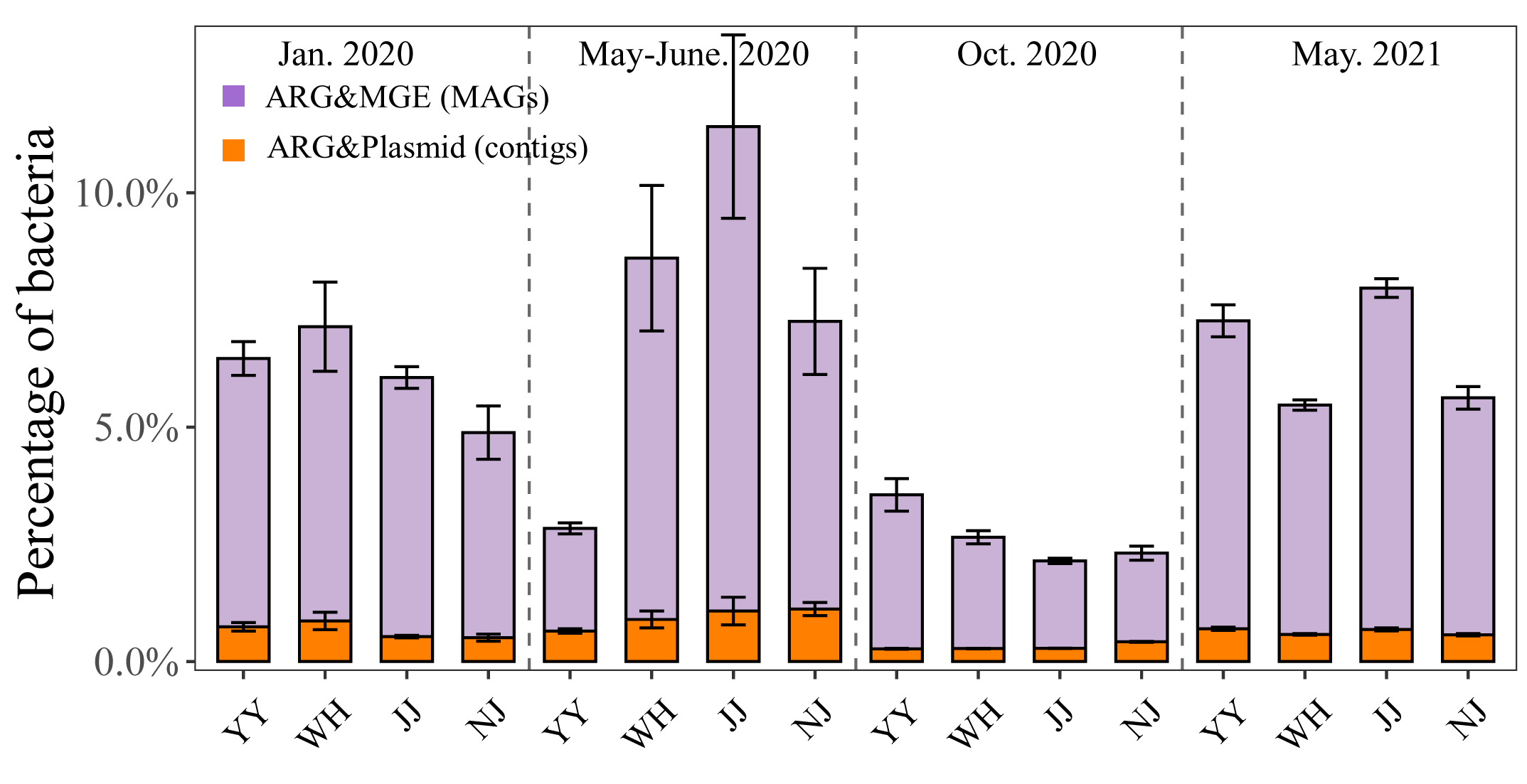


**Supplementary Figure 5. Relative abundance of MAGs carrying both ARGs and MGEs in MAG genome or MAGs with ARGs and plasmid coexistence in the same contig among reaches and times.**


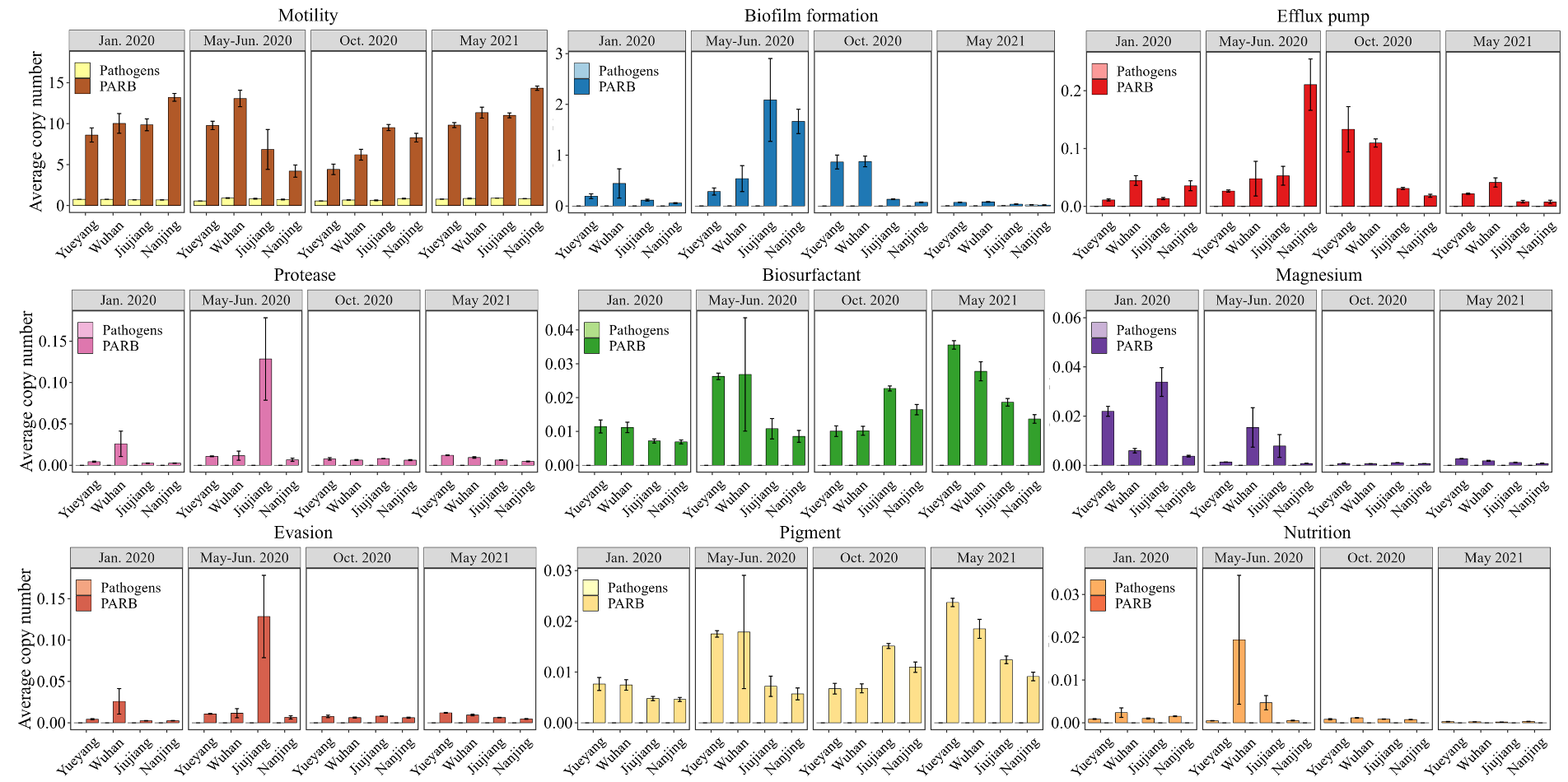


**Supplementary Figure 6. Abundance (copy number/cell) of top 9 virulence factor gene (VFG) types among reaches and times.** The Pathogens here is defined as bacteria containing VFG(s) without ARG(s), pathogenetic and antimicrobial resistant bacteria (PARB) is defined as bacterial containing both ARG(s) and VFG(s). The calculated formulas of copy number of pathogens and PARB in specific VF type were follows:

$\text{VFG}_{\text{Pathogens\_copies}}=\frac{\sum_{i=1}^{n} Pathogen_{i}\times VFGtype_{i}}{\sum_{i=1}^{n} Pathogen_{i}}$, $\text{VFG}_{\text{PARBcopies}}=\frac{\sum_{i=1}^{k} PARB_{i}\times VFGtype_{i}}{\sum_{i=1}^{n} PARB_{i}}$, where *Pathogen_i_* is the relative abundance of Pathogen *i* in the sample, *VFGtype_i_* is number of specific VFG type (i.e., motility, biofilm formation) copies in Pathogen *i* and *n* is the number of Pathogen in the sample. *PARB_i_* is the relative abundance of PARB *i* in the sample, *VFGtype_i_* is the number of specific VFG type copies in PARB *i* and k is the number of PARB in the sample. The motility virulence factor gene copies of PARB in Wuhan reaches were significantly higher in May-June 2020 than the other periods (Wilcoxon test, p < 0.05).

**Supplementary Table 1: Sampling site basic information.**

| **Time** | **Reaches** | **Sample time** | **Temperature**  **℃** | **Wind speed (m/s)** | **Precipitation (mm)** | **Humidity**  **(%)** |
| --- | --- | --- | --- | --- | --- | --- |
| January 2020 | Yueyang | 2020.1.08-1.12 | 4.1 | 2.83 | 7.7 | 89.95 |
|  | Wuhan | 2020.1.13-1.15 | 4.1 | 2.8 | 3.2 | 72.98 |
|  | Jiujiang | 2020.1.04-1.06 | 8.7 | 2.88 | 0.2 | 94.26 |
|  | Nanjing | 2020.1.01-1.03 | 6.9 | 1.9 | 0.3 | 86.85 |
| May-June 2020 | Yueyang | 2020.06.29-6.30 | 26.1 | 2.01 | 9.4 | 98.53 |
|  | Wuhan | 2020.05.01-5.03 | 26.9 | 3.05 | 0 | 68.12 |
|  | Jiujiang | 2020.06.26-6.27 | 27.6 | 2.24 | 9.2 | 89.47 |
|  | Nanjing | 2020.06.17-6.24 | 23.8 | 2.72 | 5.2 | 90.05 |
| October 2020 | Yueyang | 2020.10.17 | 15.6 | 4.6 | 2.3 | 99.64 |
|  | Wuhan | 2020.10.18 | 15.4 | 1.34 | 0 | 76.77 |
|  | Jiujiang | 2020.10.20-10.21 | 16 | 1.9 | 2.7 | 84.49 |
|  | Nanjing | 2020.10.23-10.24 | 12.8 | 2.03 | 0 | 59.34 |
| May 2021 | Yueyang | 2021.05.22 | 19.2 | 1.6 | 47.5 | 100 |
|  | Wuhan | 2021.05.17-05.20 | 19.4 | 1.81 | 0.1 | 84.94 |
|  | Jiujiang | 2021.05.25 | 24.8 | 2 | 0 | 70.03 |
|  | Nanjing | 2021.05.28 | 24.5 | 2.37 | 8.3 | 70.95 |

**Supplementary Table 2: Environment variables of 186 samples.**

| **Sample** | **T (℃)** | **pH** | **ORP (mv)** | **EC (μs/cm)** | **DO (mg/L)** | **Residual Cl (mg/L)** | **TOC (mg/L)** | | **TN (mg/L)** | **TP (mg/L)** | **Longitude E** | **Latitude N** |
| --- | --- | --- | --- | --- | --- | --- | --- | --- | --- | --- | --- | --- |
| WH1_5_20 | 20.8 | 8.31 | NA | 350 | 8.91 | 0.03 | | 1.95 | 1.78 | 0.056 | 114.00917 | 30.27194 |
| WH2_5_20 | 20.9 | 8.27 | NA | 350 | 8.89 | 0.04 | | 2.03 | 1.77 | 0.06 | 114.11556 | 30.3475 |
| WH3_5_20 | 20.7 | 8.25 | NA | 346 | 8.91 | 0.03 | | 2.09 | 1.75 | 0.053 | 114.17 | 30.40972 |
| WH4_5_20 | 20.5 | 8.26 | NA | 346 | 9.01 | 0.03 | | 2 | 1.67 | 0.054 | 114.23167 | 30.48194 |
| WH5_5_20 | 20.5 | 8.29 | NA | 348 | 9.25 | 0.02 | | 2.1 | 1.82 | 0.057 | 114.27361 | 30.5275 |
| WH6_5_20 | 20.5 | 8.24 | NA | 342.5 | 8.85 | 0.02 | | 1.87 | 1.76 | 0.062 | 114.29861 | 30.57278 |
| WH7_5_20 | 20.2 | 8.22 | NA | 339.9 | 8.79 | 0.03 | | 1.92 | 1.7 | 0.053 | 114.32556 | 30.60944 |
| WH8_5_20 | 20.5 | 8.27 | NA | 335.7 | 8.95 | 0.03 | | 1.99 | 1.67 | 0.053 | 114.35917 | 30.6475 |
| WH9_5_20 | 19.8 | 8.22 | NA | 335.5 | 8.86 | 0.01 | | 2.01 | 1.8 | 0.064 | 114.47444 | 30.6825 |
| WH10_5_20 | 20.2 | 8.24 | NA | 334.1 | 8.79 | 0.01 | | 2.22 | 1.79 | 0.073 | 114.55778 | 30.6325 |
| WH11_5_20 | 19.8 | 8.17 | NA | 329.8 | 8.95 | 0.02 | | 2.17 | 1.75 | 0.058 | 114.71694 | 30.59028 |
| H1_5_20 | 22 | 8.29 | NA | 310.4 | 8.47 | 0.02 | | NA | NA | NA | 114.23833 | 30.57056 |
| H2_5_20 | 22.2 | 8.3 | NA | 316 | 8.64 | 0.04 | | NA | NA | NA | 114.19528 | 30.58444 |
| H3_5_20 | 22.4 | 8.31 | NA | 320.1 | 8.51 | NA | | NA | NA | NA | #N/A | #N/A |
| YY1_6_20 | 24.9 | 7.65 | 259.6 | 248 | 6.87 | NA | | 2.546 | 2.4 | 0.061666667 | 113.082222 | 29.38944 |
| YY2_6_20 | 26.1 | 7.81 | 247.3 | 292 | 6.45 | NA | | 3.141 | 2.5 | 0.03 | 113.13583 | 29.44583 |
| YY3_6_20 | 24.9 | 7.94 | 191.8 | 337 | 7.15 | NA | | 2.347 | 2.6 | 0.043333333 | 113.21722 | 29.54417 |
| YY4_6_20 | 25 | NA | 165.1 | 324 | 6.95 | NA | | 2.556 | 2.6 | 0.046666667 | 113.268611 | 29.59444 |
| YY5_6_20 | 26.1 | NA | 157 | 322 | 7.03 | NA | | 2.503 | 2.2 | 0.08 | 113.33417 | 29.66556 |
| YY6_6_20 | 25.4 | NA | 160.4 | 320 | 7.14 | NA | | 2.534 | 2.2 | 0.083333333 | 113.42056 | 29.75194 |
| YY7_6_20 | 25.6 | NA | 138.1 | 338 | 7.48 | NA | | 1.949 | 2.3 | 0.036666667 | 113.49111 | 29.79778 |
| YY8_6_20 | 25.5 | NA | 164.8 | 311 | 7.73 | NA | | 2.44 | 2.6 | 0.066666667 | 113.56417 | 29.85111 |
| YY9_6_20 | 25.2 | NA | 296.1 | 327 | 7.87 | NA | | 2.536 | 2 | 0.033333333 | 113.64167 | 29.91278 |
| YY10_6_20 | 26.1 | NA | 209.2 | 315 | 7.78 | NA | | 2.545 | 2.8 | 0.09 | 113.72528 | 29.92194 |
| YY11_6_20 | 25.6 | NA | 163.3 | 343 | 8.14 | NA | | 2.554 | 3.6 | 0.093333333 | 113.81944 | 29.93417 |

**Supplementary Table 2 continued-1.**

| **Sample** | **T (℃)** | **pH** | **ORP (mv)** | **EC (μs/cm)** | **DO (mg/L)** | **Residual Cl (mg/L)** | **TOC (mg/L)** | **TN (mg/L)** | **TP (mg/L)** | **Longitude E** | **Latitude N** |
| --- | --- | --- | --- | --- | --- | --- | --- | --- | --- | --- | --- |
| JJ1_6_20 | 28 | 7.21 | 200.5 | 117.7 | 7.12 | NA | 2.53 | 1.2 | 0.018333 | 116.21444 | 29.74972 |
| JJ2_6_20 | 26.7 | 7.64 | 169.8 | 330 | 6.51 | NA | 2.392 | 1.2 | 0.025 | 116.26944 | 29.7875 |
| JJ3_6_20 | 25.6 | 7.66 | 153.4 | 329 | 6.53 | NA | 2.25 | 1.4 | 0.055 | 116.35361 | 29.84333 |
| JJ4_6_20 | 25.4 | 7.72 | 209.7 | 334 | 6.59 | NA | 3.165 | 1.5 | 0.088333 | 116.465 | 29.89472 |
| JJ5_6_20 | 26 | 7.7 | 159.9 | 341 | 6.74 | NA | 2.893 | 1.3 | 0.098333 | 116.55611 | 29.92556 |
| JJ7_6_20 | 26.3 | 7.69 | 287.9 | 323 | 6.73 | NA | 2.57 | 1.3 | 0.078333 | 116.73278 | 30.04472 |
| JJ8_6_20 | 26 | 7.92 | 224.2 | 327 | 6.9 | NA | 2.843 | 1.2 | 0.075 | 116.77667 | 30.09333 |
| JJ9_6_20 | 26.1 | 7.82 | 205.2 | 325 | 6.43 | NA | 2.656 | 1.4 | 0.078333 | 116.86472 | 30.16917 |
| JJ10_6_20 | 26.7 | 7.86 | 173.5 | 326 | 6.73 | NA | 2.047 | 1.1 | 0.055 | 116.92278 | 30.27722 |
| JJ11_6_20 | 26.7 | 7.95 | 181.5 | 330 | 6.46 | NA | 2.883 | 1.2 | 0.055 | 116.90222 | 30.34444 |
| NJ1_6_20 | 27.6 | 7.73 | 216.7 | 312 | 6.34 | NA | 2.251 | 1.6 | 0.07 | 118.49833 | 31.78944 |
| NJ2_6_20 | 27.5 | 7.69 | 232.3 | 278 | 6.09 | NA | 2.49 | 0.9 | 0.08 | 118.555 | 31.88917 |
| NJ3_6_20 | 27.5 | 7.77 | 253.4 | 325 | 6.25 | NA | 2.447 | 1.1 | 0.08 | 118.63833 | 31.96667 |
| NJ4_6_20 | 27.4 | 7.82 | 222.7 | 278 | 5.99 | NA | 2.48 | 0.9 | 0.096667 | 118.66083 | 32.00917 |
| NJ5_6_20 | 27.5 | 7.92 | 240.2 | 277 | 6.02 | NA | 2.574 | 1.3 | 0.09 | 118.69139 | 32.04361 |
| NJ6_6_20 | 27.7 | 7.94 | 231.6 | 276 | 6.66 | NA | 2.929 | 1.3 | 0.1 | 118.71194 | 32.06528 |
| NJ7_6_20 | 25.5 | 7.99 | 201.4 | 284 | 6.45 | NA | 2.74 | 1.2 | 0.1 | 118.735 | 32.10472 |
| NJ8_6_20 | 25.6 | 7.97 | 218.7 | 290 | 6.4 | NA | 2.237 | 1.1 | 0.11 | 118.74778 | 32.11639 |
| NJ9_6_20 | 25.9 | 8.04 | 233.1 | 295 | 6.44 | NA | 2.105 | 1.3 | 0.1 | 118.79389 | 32.14667 |
| NJ10_6_20 | 26.1 | 7.67 | 243.4 | 295 | 6.47 | NA | 2.671 | 1.2 | 0.056667 | 118.76028 | 32.20694 |
| NJ11_6_20 | 25.9 | 7.9 | 202.9 | 306 | 6.38 | NA | 2.73 | 1.2 | 0.07 | 118.80306 | 32.23472 |
| NJ12_6_20 | 26 | 7.97 | 211.6 | 289 | 6.58 | NA | 2.355 | 1.4 | 0.113333 | 118.87333 | 32.18194 |
| NJ13_6_20 | 26.2 | 7.97 | 208 | 294 | 6.54 | NA | 2.828 | 0.9 | 0.063333 | 119.05167 | 32.21972 |
| NJ14_6_20 | 26 | 9.87 | 188.8 | 289 | 6.55 | NA | 2.655 | 1 | 0.073333 | 119.21972 | 32.21972 |

**Supplementary Table 2 continued-2.**

| **Sample** | **T (℃)** | **pH** | **ORP (mv)** | **EC (μs/cm)** | **DO (mg/L)** | **Residual Cl (mg/L)** | **TOC (mg/L)** | **TN (mg/L)** | **TP (mg/L)** | **Longitude E** | **Latitude N** |
| --- | --- | --- | --- | --- | --- | --- | --- | --- | --- | --- | --- |
| YY1_1_20 | 9.618 | 8.781 | 146.5 | 305.1 | 11.459 | NA | 2.37 | 1.7 | 0.23 | 113.08222 | 29.38944 |
| YY2_1_20 | 9.097 | 8.361 | 268.6 | 309.8 | 12.049 | NA | 2.573 | 1.8 | 0.25 | 113.13583 | 29.44583 |
| YY3_1_20 | 9.934 | 8.635 | 227.4 | 358.9 | 11.239 | NA | 2.242 | 1.6 | 0.26 | 113.21722 | 29.54417 |
| YY4_1_20 | 9.917 | 8.694 | 150.7 | 343.3 | 12.463 | NA | 2.719 | 1.8 | 0.22 | 113.26861 | 29.59444 |
| YY5_1_20 | 9.79 | 8.621 | 245.4 | 359.2 | 11.936 | NA | 2.079 | 1.6 | 0.26 | 113.33417 | 29.66556 |
| YY6_1_20 | 9.729 | 8.41 | 256.1 | 354.6 | 12.049 | NA | 2.206 | 1.5 | 0.24 | 113.42056 | 29.75194 |
| YY7_1_20 | 9.74 | 8.645 | 255.5 | 365.6 | 12.019 | NA | 2.837 | 1.7 | 0.25 | 113.49111 | 29.79778 |
| YY8_1_20 | 9.766 | 8.472 | 247.7 | 355.7 | 12.352 | NA | 2.027 | 2 | 0.28 | 113.56417 | 29.85111 |
| YY9_1_20 | 9.976 | 8.713 | 192.2 | 361.8 | 11.838 | NA | 2.689 | 1.8 | 0.27 | 113.64167 | 29.91278 |
| YY10_1_20 | 9.955 | 8.71 | 225.9 | 347.5 | 12.075 | NA | 2.654 | 1.9 | 0.25 | 113.72528 | 29.92194 |
| WH1_1_20 | 11.185 | 8.697 | 251.9 | 350.5 | 11.148 | NA | 2.118 | 1.7 | 0.22 | 114.00917 | 30.27194 |
| WH2_1_20 | 11.21 | 8.549 | 230.1 | 350.9 | 11.048 | NA | 2.065 | 1.6 | 0.27 | 114.11556 | 30.3475 |
| WH3_1_20 | 11.511 | 8.664 | 229.3 | 350.5 | 11.02 | NA | 1.981 | 2 | 0.25 | 114.17 | 30.40972 |
| WH4_1_20 | 10.413 | 8.609 | 194.7 | 380.5 | 11.706 | NA | 2.562 | 2.1 | 0.27 | 114.23167 | 30.48194 |
| WH6_1_20 | 9.02 | 8.886 | 154.6 | 364.3 | 12.495 | NA | 2.664 | 2 | 0.24 | 114.29861 | 30.57278 |
| WH7_1_20 | 12.34 | 8.49 | 240.9 | 348.9 | 10.77 | NA | 2.157 | 1.9 | 0.25 | 114.32556 | 30.60944 |
| WH8_1_20 | 11.505 | 8.444 | 236.5 | 342.8 | 10.54 | NA | 2.635 | 1.9 | 0.2 | 114.35917 | 30.6475 |
| WH9_1_20 | 11.83 | 8.515 | 208 | 355.3 | 10.705 | NA | 2.454 | 1.8 | 0.17 | 114.47444 | 30.6825 |
| WH10_1_20 | 12.491 | 8.556 | 224.4 | 349 | 10.478 | NA | 2.037 | 1.6 | 0.29 | 114.55778 | 30.6325 |
| WH11_1_20 | 12.606 | 8.969 | 201 | 355.4 | 10.755 | NA | 2.291 | 1.7 | 0.22 | 114.71694 | 30.59028 |
| H2_1_20 | 7.634 | 8.697 | 197 | 327.6 | 12.38 | NA | NA | NA | NA | 114.19528 | 30.58444 |
| JJ1_1_20 | 11.9 | 7.49 | 160.2 | 327.9 | 9.91 | NA | 5.194 | 1.6 | 0.12 | 116.21444 | 29.74972 |
| JJ2_1_20 | 12.4 | 8.032 | 154.7 | 325.2 | 9.58 | NA | 4.859 | 2 | 0.26 | 116.26944 | 29.7875 |
| JJ3_1_20 | 12.5 | 8.542 | 146.3 | 330.6 | 9.62 | NA | 4.01 | 1.8 | 0.24 | 116.35361 | 29.84333 |

**Supplementary Table 2 continued-3.**

| **Sample** | **T (℃)** | **pH** | **ORP (mv)** | **EC (μs/cm)** | **DO (mg/L)** | **Residual Cl (mg/L)** | **TOC (mg/L)** | **TN (mg/L)** | **TP (mg/L)** | **Longitude E** | **Latitude N** |
| --- | --- | --- | --- | --- | --- | --- | --- | --- | --- | --- | --- |
| JJ4_1_20 | 12.8 | 8.259 | 154.8 | 320.4 | 9.74 | NA | 4.105 | 1.9 | 0.22 | 116.465 | 29.89472 |
| JJ5_1_20 | 11.89 | 8.714 | 148.5 | 330.3 | 11.156 | NA | 3.148 | 1.8 | 0.27 | 116.55611 | 29.92556 |
| JJ6_1_20 | 13.7 | 8.726 | 144.9 | 324.2 | 10.121 | NA | 2.742 | 1.9 | 0.29 | 116.62694 | 29.97917 |
| JJ7_1_20 | 14.24 | 8.662 | 135.2 | 320.7 | 9.847 | NA | 3.237 | 1.8 | 0.29 | 116.73278 | 30.04472 |
| JJ8_1_20 | 13.078 | 9.525 | 160.7 | 322.9 | 9.719 | NA | 2.98 | 1.6 | 0.28 | 116.77667 | 30.09333 |
| JJ9_1_20 | 13.597 | 9.7 | 120.7 | 320.5 | 9.949 | NA | 1.72 | 2 | 0.29 | 116.86472 | 30.16917 |
| JJ10_1_20 | 14.241 | 8.657 | 111.2 | 310.6 | 9.674 | NA | 2.42 | 1.9 | 0.25 | 116.92278 | 30.27722 |
| JJ11_1_20 | 15.19 | 8.724 | 112.6 | 324.6 | 9.696 | NA | 2.41 | 1.8 | 0.27 | 116.90222 | 30.34444 |
| NJ1_1_20 | 12.94 | 8.22 | 216.6 | 335.1 | 10.671 | NA | 2.545 | 2.1 | 0.16 | 118.49833 | 31.78944 |
| NJ2_1_20 | 12.751 | 8.189 | 219.9 | 334.6 | 10.902 | NA | 4.569 | 1.8 | 0.15 | 118.555 | 31.88917 |
| NJ3_1_20 | 12.462 | 8.144 | 243.1 | 335.7 | 10.945 | NA | 4.26 | 2.4 | 0.16 | 118.63833 | 31.96667 |
| NJ4_1_20 | 12.277 | 8.137 | 232.4 | 334.6 | 11.287 | NA | 4.3 | 2.3 | 0.13 | 118.66083 | 32.00917 |
| NJ5_1_20 | 9.231 | 8.587 | 177.5 | 341.5 | 12.817 | NA | 4.42 | 2.30 | 0.26 | 118.69139 | 32.04361 |
| NJ6_1_20 | 10.383 | 8.457 | 213.2 | 342.5 | 12.163 | NA | 3.73 | 2.60 | 0.20 | 118.71194 | 32.06528 |
| NJ7_1_20 | 10.717 | 8.162 | 213.4 | 337.4 | 11.807 | NA | 4.42 | 2.60 | 0.15 | 118.735 | 32.10472 |
| NJ8_1_20 | 9.002 | 8.249 | 237.6 | 353.1 | 12.66 | NA | 4.30 | 1.90 | 0.15 | 118.74778 | 32.11639 |
| NJ9_1_20 | 10.487 | 8.482 | 235.7 | 338.4 | 11.86 | NA | 4.21 | 2.10 | 0.27 | 118.79389 | 32.14667 |
| NJ10_1_20 | 11.244 | 8.067 | 262.6 | 345 | 12.634 | NA | 4.22 | 2.10 | 0.24 | 118.76028 | 32.20694 |
| NJ11_1_20 | 10.014 | 8.339 | 245.1 | 341 | 11.868 | NA | 4.19 | 2.20 | 0.29 | 118.80306 | 32.23472 |
| NJ12_1_20 | 10.008 | 8.62 | 215.3 | 348.9 | 12.362 | NA | 4.37 | 2.20 | 0.26 | 118.87333 | 32.18194 |
| NJ13_1_20 | 10.732 | 8.705 | 151 | 338.2 | 11.747 | NA | 7.17 | 2.10 | 0.24 | 119.05167 | 32.21972 |
| NJ14_1_20 | 10.951 | 8.574 | 210.1 | 336.9 | 11.791 | NA | 7.48 | 1.90 | 0.27 | 119.21972 | 32.21972 |
| YY1_10_20 | 19 | 7.58 | 216.7 | 265 | 8.54 | NA | 2.597 | 3.296901 | 0.068848 | 113.08222 | 29.38944 |
| YY2_10_20 |  | 8.02 | 199.1 | 293 | 8.82 | NA | 2.476 | 2.462574 | 0.037266 | 113.13583 | 29.44583 |

**Supplementary Table 2 continued-4.**

| **Sample** | **T (℃)** | **pH** | **ORP (mv)** | **EC (μs/cm)** | **DO (mg/L)** | **Residual Cl (mg/L)** | **TOC (mg/L)** | **TN (mg/L)** | **TP (mg/L)** | **Longitude E** | **Latitude N** |
| --- | --- | --- | --- | --- | --- | --- | --- | --- | --- | --- | --- |
| YY3_10_20 | 20.6 | 7.85 | 220.4 | 331 | 8.7 | NA | 2.435 | 2.28379 | 0.027792 | 113.21722 | 29.54417 |
| YY4_10_20 | 18.6 | 7.93 | 211.1 | 330 | 8.54 | NA | 2.584 | 1.509058 | 0.056215 | 113.26861 | 29.59444 |
| YY5_10_20 | 20.3 | 7.83 | 199.7 | 317 | 8.37 | NA | 2.683 | 1.270679 | 0.03095 | 113.33417 | 29.66556 |
| YY6_10_20 | 19.7 | 7.75 | 201.1 | 330 | 8.8 | NA | 2.289 | 4.250417 | 0.034108 | 113.42056 | 29.75194 |
| YY7_10_20 | 19.7 | 7.86 | 224.5 | 321 | 8.43 | NA | 2.298 | 2.522169 | 0.03095 | 113.49111 | 29.79778 |
| YY8_10_20 | 20.1 | 7.7 | 209.6 | 308 | 8.48 | NA | 2.245 | 2.462574 | 0.043583 | 113.56417 | 29.85111 |
| YY9_10_20 | 20.1 | 7.82 | 233 | 310 | 8.21 | NA | 3.131 | 2.939333 | 0.03095 | 113.64167 | 29.91278 |
| YY10_10_20 | 20.1 | 7.81 | 217.4 | 296 | 8.32 | NA | 2.288 | 2.998927 | 0.040424 | 113.72528 | 29.92194 |
| YY11_10_20 |  | 7.6 | 209.8 | 312 | 8.71 | NA | 3.418 | 0.913111 | 0.027792 | 113.81944 | 29.93417 |
| WH1_10_20 | 19.7 | 7.96 | 197.4 | 315 | 8.4 | NA | 2.506 | 2.462574 | 0.037266 | 114.00917 | 30.27194 |
| WH2_10_20 | 20.2 | 7.7 | 227.9 | 317 | 8.36 | NA | 2.545 | 1.389869 | 0.03095 | 114.11556 | 30.3475 |
| WH3_10_20 | 20.1 | 7.76 | 203.7 | 314 | 8.54 | NA | 3.15 | 1.985816 | 0.043583 | 114.17 | 30.40972 |
| WH4_10_20 | 20.4 | 7.87 | 208 | 312 | 8.35 | NA | 3.532 | 1.866627 | 0.049899 | 114.23167 | 30.48194 |
| WH5_10_20 | 20.2 | 8.07 | 225.4 | 312 | 8.4 | NA | 3.102 | 1.71764 | 0.043583 | 114.27361 | 30.5275 |
| WH6_10_20 | 19.7 | 7.81 | 225.8 | 316 | 8.61 | NA | 3.36 | 1.985816 | 0.027792 | 114.29861 | 30.57278 |
| WH7_10_20 | 20.2 | 7.85 | 229.1 | 313 | 8.42 | NA | 3.195 | 1.866627 | 0.043583 | 114.32556 | 30.60944 |
| WH8_10_20 | 20 | 7.89 | 249.9 | 315 | 8.44 | NA | 2.926 | 1.389869 | 0.043583 | 114.35917 | 30.6475 |
| WH9_10_20 | 20.7 | 7.84 | 250.6 | 312 | 8.37 | NA | 2.986 | 2.700954 | 0.037266 | 114.47444 | 30.6825 |
| WH10_10_20 | 20.6 | 7.88 | 248.1 | 312 | 8.22 | NA | 3.153 | 1.628248 | 0.040424 | 114.55778 | 30.6325 |
| WH11_10_20 | 20.1 | 7.82 | 229.9 | 319 | 8.31 | NA | 3.508 | 0.913111 | 0.06569 | 114.71694 | 30.59028 |
| JJ1_10_20 | 19 | 7.39 | 206.8 | 145.2 | 8.62 | NA | 3.047 | 1.008462 | 0.056015 | 116.21444 | 29.74972 |
| JJ2_10_20 | 19.6 | NA | 215.5 | 298 | 8.19 | NA | 2.93 | 1.961979 | 0.049899 | 116.26944 | 29.7875 |
| JJ3_10_20 | 20.4 | 7.81 | 247.1 | 321 | 8.2 | NA | 2.814 | 2.081168 | 0.049899 | 116.35361 | 29.84333 |
| JJ4_10_20 | 19.9 | 7.52 | 205.7 | 309 | 8.28 | NA | 3.054 | 2.200358 | 0.037266 | 116.465 | 29.89472 |

**Supplementary Table 2 continued-5.**

| **Sample** | **T (℃)** | **pH** | **ORP (mv)** | **EC (μs/cm)** | **DO (mg/L)** | **Residual Cl (mg/L)** | **TOC (mg/L)** | **TN (mg/L)** | **TP (mg/L)** | **Longitude E** | **Latitude N** |
| --- | --- | --- | --- | --- | --- | --- | --- | --- | --- | --- | --- |
| JJ5_10_20 | 20 | 7.77 | 209.2 | 291 | 8.15 | NA | 2.538 | 2.081168 | 0.049899 | 116.55611 | 29.92556 |
| JJ6_10_20 | 20.3 | 7.75 | 221.4 | 294 | 8.1 | NA | 1.925 | 1.628248 | 0.034108 | 116.62694 | 29.97917 |
| JJ7_10_20 | 20.7 | 7.78 | 226.6 | 299 | 8.36 | NA | 2.959 | 2.105006 | 0.059373 | 116.73278 | 30.04472 |
| JJ8_10_20 | 20.2 | 7.74 | 225.9 | 301 | 8.07 | NA | 2.748 | 1.747437 | 0.034108 | 116.77667 | 30.09333 |
| JJ9_10_20 | 20.3 | 7.75 | 240.5 | 295 | 7.92 | NA | 2.085 | 2.40298 | 0.034108 | 116.86472 | 30.16917 |
| JJ10_10_20 | 20.5 | 7.74 | 211.6 | 298 | 7.96 | NA | 2.687 | 1.985816 | 0.049899 | 116.92278 | 30.27722 |
| JJ11_10_20 |  | NA | 252 | 299 | 8.23 | NA | 2.826 | 3.65447 | 0.056215 | 116.90222 | 30.34444 |
| NJ1_10_20 | 20.6 | 7.64 | 217.9 | 297 | 8.11 | NA | 3.516 | 2.522169 | 0.072006 | 118.49833 | 31.78944 |
| NJ2_10_20 | 20 | 7.68 | 218.5 | 300 | 8.24 | NA | 2.862 | 1.687843 | 0.125695 | 118.555 | 31.88917 |
| NJ3_10_20 | 20.3 | 7.76 | 235.9 | 296 | 8.01 | NA | 2.974 | 1.926222 | 0.075164 | 118.63833 | 31.96667 |
| NJ4_10_20 | 19.9 | 7.75 | 234.4 | 297 | 8.21 | NA | 3.601 | 2.641359 | 0.078322 | 118.66083 | 32.00917 |
| NJ5_10_20 | 20.4 | 7.78 | 247.4 | 295 | 8.25 | NA | 2.911 | 1.479261 | 0.062532 | 118.69139 | 32.04361 |
| NJ6_10_20 | 19.8 | 7.76 | 248.1 | 295 | 8.35 | NA | 3.142 | 1.747437 | 0.075164 | 118.71194 | 32.06528 |
| NJ7_10_20 | 19.9 | 7.73 | 252.6 | 296 | 8.02 | NA | 3.015 | 1.509058 | 0.084639 | 118.735 | 32.10472 |
| NJ8_10_20 | 20.2 | 7.68 | 261 | 303 | 8.09 | NA | 2.788 | 1.82886 | 0.034108 | 118.74778 | 32.11639 |
| NJ9_10_20 | 20.3 | 7.64 | 225.3 | 296 | 8.12 | NA | 2.922 | 1.747437 | 0.027792 | 118.79389 | 32.14667 |
| NJ10_10_20 | 20.4 | 7.51 | 231.7 | 296 | 8.41 | NA | 2.818 | 1.866627 | 0.056215 | 118.76028 | 32.20694 |
| NJ12_10_20 | 19.5 | 7.79 | 298.1 | 295 | 8.61 | NA | 3.888 | 1.687843 | 0.087797 | 118.87333 | 32.18194 |
| NJ13_10_20 | 19.7 | 7.7 | 282.2 | 300 | 8.32 | NA | 3.933 | 1.747437 | 0.053057 | 119.05167 | 32.21972 |
| NJ14_10_20 | 19.9 | 7.72 | 255.9 | 295 | 8.32 | NA | 4.009 | 1.628248 | 0.03095 | 119.21972 | 32.21972 |
| YY1_5_21 | 21.91 | 7.993 | 192.4 | 242.9 | 8.182 | NA | 1.971 | 1.638458 | 0.133 | 113.08222 | 29.38944 |
| YY2_5_21 | 22.15 | 8.004 | 187.9 | 243.4 | 8.472 | NA | 2.069 | 1.638458 | 0.0935 | 113.13583 | 29.44583 |
| YY3_5_21 | 22.17 | 8.079 | 178.4 | 318.5 | 8.376 | NA | 1.981 | 1.619243 | 0.14375 | 113.21722 | 29.54417 |
| YY4_5_21 | 22.12 | 8.108 | 172 | 331.4 | 8.385 | NA | 1.933 | 1.66728 | 0.154 | 113.26861 | 29.59444 |

**Supplementary Table 2 continued-6.**

| **Sample** | **T (℃)** | **pH** | **ORP (mv)** | **EC (μs/cm)** | **DO (mg/L)** | **Residual Cl (mg/L)** | **TOC (mg/L)** | **TN (mg/L)** | **TP (mg/L)** | **Longitude E** | **Latitude N** |
| --- | --- | --- | --- | --- | --- | --- | --- | --- | --- | --- | --- |
| YY5_5_21 | 22.1 | 7.911 | 164.6 | 335 | 8.631 | NA | 1.81 | 1.628851 | 0.1045 | 113.33417 | 29.66556 |
| YY6_5_21 | 22.4 | 7.996 | 152.2 | 279 | 8.455 | NA | 2.036 | 1.753745 | 0.193 | 113.42056 | 29.75194 |
| YY7_5_21 | 22.45 | 7.991 | 148.9 | 347.9 | 8.321 | NA | 1.786 | 1.715316 | 0.16275 | 113.49111 | 29.79778 |
| YY8_5_21 | 22.51 | 8.043 | 140.2 | 396.7 | 8.342 | NA | NA | NA | NA | 113.56417 | 29.85111 |
| YY9_5_21 | 22.58 | 8.023 | 138.5 | 337 | 8.289 | NA | NA | NA | NA | 113.64167 | 29.91278 |
| YY10_5_21 | 22.62 | 7.968 | 128.3 | 332.1 | 8.295 | NA | NA | NA | NA | 113.72528 | 29.92194 |
| YY11_5_21 | 22.55 | 7.979 | 124.2 | 395 | 8.325 | NA | NA | NA | NA | 113.81944 | 29.93417 |
| WH1_5_21 | 22.78 | 8.302 | 153 | 310.1 | 8.112 | NA | 1.414 | 1.551992 | 0.05525 | 114.00917 | 30.27194 |
| WH2_5_21 | 22.79 | 8.319 | 149.4 | 314.4 | 8.014 | NA | 1.386 | 1.494348 | 0.05025 | 114.11556 | 30.3475 |
| WH3_5_21 | 22.71 | 8.332 | 144.5 | 319 | 8.221 | NA | 1.478 | 1.503956 | 0.04825 | 114.17 | 30.40972 |
| WH4_5_21 | 22.66 | 8.333 | 142.3 | 318.6 | 8.076 | NA | 1.453 | 1.436705 | 0.0385 | 114.23167 | 30.48194 |
| WH5_5_21 | 22.65 | 8.315 | 137.6 | 324.5 | 8.254 | NA | 1.588 | 1.503956 | 0.03725 | 114.27361 | 30.5275 |
| WH6_5_21 | 22.4 | 8.293 | 135.5 | 330 | 8.152 | NA | 1.523 | 1.571207 | 0.092 | 114.29861 | 30.57278 |
| WH7_5_21 | 23.42 | 8.21 | 94.59 | 335.3 | 8.06 | NA | NA | NA | NA | 114.32556 | 30.60944 |
| WH8_5_21 | 23.23 | 8.235 | 103.3 | 331.8 | 8.199 | NA | NA | NA | NA | 114.35917 | 30.6475 |
| WH9_5_21 | 23.25 | 8.22 | 104.7 | 330.1 | 8.267 | NA | NA | NA | NA | 114.47444 | 30.6825 |
| WH10_5_21 | 23.35 | 8.244 | 107.8 | 329 | 8.297 | NA | NA | NA | NA | 114.55778 | 30.6325 |
| WH11_5_21 | 23.45 | 8.246 | 108.2 | 329 | 8.345 | NA | NA | NA | NA | 114.71694 | 30.59028 |
| JJ1_5_21 | 24.32 | 7.424 | 235.2 | 158.8 | 8.421 | NA | 2.525 | 1.41749 | 0.03075 | 116.21444 | 29.74972 |
| JJ2_5_21 | 23.81 | 7.478 | 228.2 | 275.6 | 8.052 | NA | 2.057 | 1.77296 | 0.1055 | 116.26944 | 29.7875 |
| JJ3_5_21 | 23.62 | 7.676 | 228.6 | 269.6 | 8.028 | NA | 2.051 | 1.532778 | 0.09875 | 116.35361 | 29.84333 |
| JJ4_5_21 | 23.75 | 7.567 | 229.4 | 268.9 | 8.124 | NA | 2.139 | 1.696102 | 0.0915 | 116.465 | 29.89472 |
| JJ5_5_21 | 24.05 | 7.542 | 232.2 | 285.3 | 8.046 | NA | 2.056 | 1.77296 | 0.12725 | 116.55611 | 29.92556 |
| JJ6_5_21 | 24.55 | 7.532 | 235.5 | 272 | 8.022 | NA | 2.103 | 1.77296 | 0.0965 | 116.62694 | 29.97917 |

**Supplementary Table 2 continued-7.**

| **Sample** | **T (℃)** | **pH** | **ORP (mv)** | **EC (μs/cm)** | **DO (mg/L)** | **Residual Cl (mg/L)** | **TOC (mg/L)** | **TN (mg/L)** | **TP (mg/L)** | **Longitude E** | **Latitude N** |
| --- | --- | --- | --- | --- | --- | --- | --- | --- | --- | --- | --- |
| JJ7_5_21 | 24.58 | 7.555 | 239.4 | 271.5 | 8.065 | NA | 2.122 | 1.734531 | 0.10425 | 116.73278 | 30.04472 |
| JJ8_5_21 | 24.6 | 7.698 | 241.8 | 273.2 | 7.947 | NA | 2.108 | 1.965106 | 0.13375 | 116.77667 | 30.09333 |
| JJ9_5_21 | 24.76 | 7.75 | 243.6 | 274 | 7.858 | NA | 2.063 | 1.580814 | 0.041 | 116.86472 | 30.16917 |
| JJ10_5_21 | 24.93 | 7.571 | 245.3 | 275.4 | 7.862 | NA | 2.055 | 1.657672 | 0.111 | 116.92278 | 30.27722 |
| JJ11_5_21 | 25.12 | 7.58 | 244.6 | 281.3 | 7.924 | NA | 2.08 | 1.628851 | 0.082 | 116.90222 | 30.34444 |
| NJ1_5_21 | 25.88 | 7.872 | 166.2 | 260.1 | 8.121 | NA | 2.196 | 1.676887 | 0.08925 | 118.49833 | 31.78944 |
| NJ2_5_21 | 25.89 | 7.839 | 168.7 | 261.9 | 8.169 | NA | 2.125 | 1.782567 | 0.104 | 118.555 | 31.88917 |
| NJ3_5_21 | 25.92 | 7.832 | 174.4 | 270.6 | 8.026 | NA | 2.132 | 1.801782 | 0.145 | 118.63833 | 31.96667 |
| NJ4_5_21 | 25.94 | 7.788 | 179.6 | 262.6 | 8.125 | NA | 2.154 | 1.696102 | 0.08425 | 118.66083 | 32.00917 |
| NJ5_5_21 | 25.94 | 7.78 | 185.5 | 264.4 | 8.142 | NA | 2.126 | 1.686494 | 0.08125 | 118.69139 | 32.04361 |
| NJ6_5_21 | 26.01 | 7.67 | 188.2 | 264.8 | 8.094 | NA | 2.064 | 1.744138 | 0.0635 | 118.71194 | 32.06528 |
| NJ7_5_21 | 26.12 | 7.602 | 192.4 | 263.7 | 8.122 | NA | 2.095 | 1.580814 | 0.07775 | 118.735 | 32.10472 |
| NJ8_5_21 | 26.1 | 7.642 | 195.2 | 264.3 | 8.056 | NA | 2.081 | 1.590421 | 0.1 | 118.74778 | 32.11639 |
| NJ9_5_21 | 26.14 | 7.569 | 198.4 | 263.8 | 8.092 | NA | 2.096 | 1.446312 | 0.06375 | 118.79389 | 32.14667 |
| NJ12_5_21 | 26.25 | 7.765 | 201.3 | 266 | 8.32 | NA | 2.058 | 1.475134 | 0.06375 | 118.87333 | 32.18194 |
| NJ13_5_21 | 26.23 | 7.615 | 203.6 | 269.3 | 8.283 | NA | 2.066 | 1.484741 | 0.07225 | 119.05167 | 32.21972 |
| NJ14_5_21 | 26.28 | 7.581 | 205.2 | 269.3 | 8.24 | NA | 2.078 | 1.446312 | 0.07425 | 119.21972 | 32.21972 |

**T**: temperature; **ORP**: oxidation reduction potential; **EC**: electrical conductivity; **DO**: dissolved oxygen;

**Residual Cl**: residual chlorine. **TOC**: total organic carbon; **TN**: total nitrogen; **TP**: total phosphorus

The longitude and latitude of each site were determined by GPS.

The ORP values in Wuhan May 2020 were not collected due to the probe mistake, and the residual chlorine was only detected in Wuhan May 2020

**Supplementary Table 3: Microbial cell density (1, 000 cells/ml).**

| **Time** | **Yueyang** | **Wuhan** | **Jiujiang** | **Nanjing** |
| --- | --- | --- | --- | --- |
| January 2020 | 29.1 ± 15 | 24.7 ± 12.8 | 48.4 ± 29.2 | 38.2 ± 34.0 |
| May-June 2020 | 87.2 ± 66.5 | 11.6 ± 4.2 | 73.2 ± 96.7 | 152.4 ± 116.4 |
| October 2020 | 641.9 ± 298.7 | 922.1 ± 266.9 | 1074.3 ± 386.8 | 1185.4 ± 459.7 |
| May 2021 | 2517.9 ± 1697.2 | 441.7 ± 239.9 | 925.4 ± 458.8 | 1045.6 ± 641.8 |

**Supplementary Table 4: Prokaryotic plankton community composition (clr) difference test (Wuhan Vs Others).**

| **Test Method** | **Time** | **Wuhan vs Yueyang** | | **Wuhan vs Jiujiang** | | **Wuhan vs Nanjing** | |
| --- | --- | --- | --- | --- | --- | --- | --- |
| **ANOSIM** |  | R | *p* | R | *p* | R | *p* |
|  | January 2020 | 0.27 | 0.001 | 0.16 | 0.001 | 0.92 | 0.001 |
|  | May-June 2020 | **0.97** | 0.001 | **0.82** | 0.001 | **0.95** | 0.001 |
|  | October 2020 | 0.11 | 0.018 | 0.33 | 0.001 | 0.37 | 0.001 |
|  | May 2021 | 0.35 | 0.001 | 0.77 | 0.001 | 0.74 | 0.001 |
| **Adonis** |  | R^2^ | *p* | R^2^ | *p* | R^2^ | *p* |
|  | January 2020 | 4.11 | 0.001 | 2.42 | 0.001 | 15.43 | 0.001 |
|  | May-June 2020 | **40.66** | 0.001 | **21.76** | 0.001 | **21.2** | 0.001 |
|  | October 2020 | 0.88 | 0.007 | 5.35 | 0.001 | 7.56 | 0.001 |
|  | May 2021 | 3.66 | 0.001 | 5.64 | 0.001 | 5.75 | 0.001 |
| **MRPP** |  | delta | *p* | delta | *p* | delta | *p* |
|  | January 2020 | 0.083 | 0.001 | 0.063 | 0.001 | 0.1 | 0.001 |
|  | May-June 2020 | **0.12** | 0.001 | **0.16** | 0.001 | **0.13** | 0.001 |
|  | October 2020 | 0.079 | 0.004 | 0.065 | 0.001 | 0.071 | 0.001 |
|  | May 2021 | 0.08 | 0.001 | 0.081 | 0.001 | 0.08 | 0.001 |

**ANOSIM:** analysis of similarities;

**Adonis**: analysis of variance using distance matrices;

**MRPP**: multiple response permutation procedure.

**Bold fonts**: The highest value of each comparison (Wuhan vs Yueyang, Wuhan vs Jinjiang, and Wuhan vs Nanjing) among four sampling time.

**Supplementary Table 5: Microeukaryotic plankton community composition difference test (Wuhan Vs Others).**

| **Test Method** | **Time** | **Wuhan vs Yueyang** | | **Wuhan vs Jiujiang** | | **Wuhan vs Nanjing** | |
| --- | --- | --- | --- | --- | --- | --- | --- |
| **ANOSIM** |  | R | *p* | R | *p* | R | *p* |
|  | January 2020 | 0.58 | 0.001 | 0.67 | 0.001 | 0.81 | 0.001 |
|  | May-June 2020 | **0.88** | 0.001 | **0.70** | 0.001 | **0.97** | 0.001 |
|  | October 2020 | 0.22 | 0.002 | 0.30 | 0.001 | 0.80 | 0.001 |
|  | May 2021 | 0.42 | 0.001 | 0.28 | 0.001 | 0.26 | 0.001 |
| **Adonis** |  | R^2^ | *p* | R^2^ | *p* | R^2^ | *p* |
|  | January 2020 | 4.70 | 0.001 | 3.73 | 0.001 | 10.10 | 0.001 |
|  | May-June 2020 | **17.12** | 0.001 | **7.14** | 0.001 | **20.12** | 0.001 |
|  | October 2020 | 2.22 | 0.004 | 2.72 | 0.001 | 6.99 | 0.001 |
|  | May 2021 | 4.34 | 0.001 | 3.11 | 0.001 | 3.28 | 0.001 |
| **MRPP** |  | delta | *p* | delta | *p* | delta | *p* |
|  | January 2020 | 0.24 | 0.001 | 0.26 | 0.001 | 0.23 | 0.001 |
|  | May-June 2020 | **0.26** | 0.001 | **0.30** | 0.003 | **0.28** | 0.001 |
|  | October 2020 | 0.16 | 0.002 | 0.22 | 0.001 | 0.20 | 0.001 |
|  | May 2021 | 0.21 | 0.001 | 0.21 | 0.001 | 0.25 | 0.001 |

**ANOSIM:** analysis of similarities;

**Adonis**: analysis of variance using distance matrices;

**MRPP**: multiple response permutation procedure.

**Bold fonts**: The highest value of each comparison (Wuhan vs Yueyang, Wuhan vs Jinjiang, and Wuhan vs Nanjing) among four sampling time.

**Supplementary Table 6: Plankton function composition difference test (Wuhan Vs Others).**

| **Test Method** | **Time** | **Wuhan vs Yueyang** | | **Wuhan vs Jiujiang** | | **Wuhan vs Nanjing** | |
| --- | --- | --- | --- | --- | --- | --- | --- |
| **ANOSIM** |  | R | *p* | R | *p* | R | *p* |
|  | January 2020 | 0.620727 | 0.002 | 0.625094 | 0.001 | 0.821295 | 0.001 |
|  | May-June 2020 | **0.996353** | 0.001 | 0.764286 | 0.001 | **0.995963** | 0.001 |
|  | October 2020 | 0.224418 | 0.001 | 0.819534 | 0.001 | 0.938377 | 0.001 |
|  | May 2021 | 0.54583 | 0.001 | **0.998197** | 0.001 | 0.688204 | 0.001 |
| **Adonis** |  | R^2^ | *p* | R^2^ | *p* | R^2^ | *p* |
|  | January 2020 | 4.621441 | 0.001 | 3.591067 | 0.001 | 5.587972 | 0.001 |
|  | May-June 2020 | **22.40627** | 0.001 | **12.51716** | 0.001 | **17.97433** | 0.001 |
|  | October 2020 | 1.408059 | 0.001 | 7.177171 | 0.001 | 6.148976 | 0.002 |
|  | May 2021 | 3.484472 | 0.001 | 6.745009 | 0.001 | 4.199978 | 0.001 |
| **MRPP** |  | delta | *p* | delta | *p* | delta | *p* |
|  | January 2020 | 0.047957 | 0.001 | 0.044611 | 0.001 | 0.045472 | 0.001 |
|  | May-June 2020 | **0.050631** | 0.001 | **0.072502** | 0.001 | **0.055088** | 0.001 |
|  | October 2020 | 0.041805 | 0.001 | 0.044531 | 0.001 | 0.044083 | 0.001 |
|  | May 2021 | 0.04698 | 0.001 | 0.044412 | 0.001 | 0.051027 | 0.001 |

**ANOSIM:** analysis of similarities;

**Adonis**: analysis of variance using distance matrices;

**MRPP**: multiple response permutation procedure.

**Bold fonts**: The highest value of each comparison (Wuhan vs Yueyang, Wuhan vs Jinjiang, and Wuhan vs Nanjing) among four sampling time.

**Supplementary Table 7-1: T-test of bacterial alpha diversity in Wuhan between May-June 2020 and other periods.**

| **Test Method** |  | **Jan-20** | **May-June 20** | **Oct-20** | **May-21** |
| --- | --- | --- | --- | --- | --- |
| **Richness** | Mean | 1416 | 1258 | 1386 | 1393 |
|  | P value (May-June 2020 vs Others) | 0.002 | \ | 0.008 | 0.006 |
| **Shannon** | Mean | 9.78 | 9.24 | 9.53 | 9.71 |
|  | P value (May-June 2020 vs Others) | 0.001 | \ | 0.039 | 0.003 |
| **Chao1** | Mean | 1678 | 1604 | 1677 | 1648 |
|  | P value (May-June 2020 vs Others) | 0.094 | \ | 0.061 | 0.256 |

**Supplementary Table 7-2: T-test of microeukaryotic alpha diversity in Wuhan between May-June 2020 and other periods**

| **Test Method** |  | **Jan-20** | **May-June-20** | **Oct-20** | **May-21** |
| --- | --- | --- | --- | --- | --- |
| **Richness** | Mean | 281 | 221 | 295 | 286 |
|  | P value (May-June 2020 vs Others) | 0.001 | \ | 0.001 | 0.001 |
| **Shannon** | Mean | 7.65 | 7.25 | 7.82 | 7.74 |
|  | P value (May-June 2020 vs Others) | 0.004 | \ | 0.001 | 0.001 |
| **Chao1** | Mean | 524 | 379 | 523 | 509 |
|  | P value (May-June 2020 vs Others) | 0.001 | \ | 0.001 | 0.001 |

**Supplementary Table 8-1: The ALDEX2 output results of the 31 identified highly abundant prokaryotic OTUs in Wuhan-May-2020 vs Others.**

| **OTU** | **rab.win.Others** | **rab.win.Wuhan** | **diff.btw** | **diff.win** | **effect** | **wi.ep** | **wi.eBH** |
| --- | --- | --- | --- | --- | --- | --- | --- |
| Nocardioides OTU178 | -2.015483481 | 4.674585588 | 6.818326087 | 2.34838096 | 2.780735 | 9.38E-10 | 3.81E-08 |
| Nocardioides OTU405 | -4.564307158 | 4.3896098 | 9.180618682 | 3.132678792 | 2.860752 | 8.56E-10 | 3.81E-08 |
| Nocardioides OTU403 | -3.372154741 | 4.928060228 | 8.236892261 | 2.641422658 | 3.099642 | 6.91E-10 | 3.81E-08 |
| Novosphingobium OTU295 | -2.77825992 | 1.917048208 | 4.76658995 | 2.473613728 | 1.775772 | 1.58E-08 | 1.2E-07 |
| Novosphingobium OTU182 | -3.863448438 | 2.652235733 | 6.491498068 | 3.384178998 | 1.805436 | 2.01E-08 | 1.43E-07 |
| Novosphingobium OTU206 | -2.976586465 | 1.835017961 | 5.089949956 | 2.536005332 | 1.844602 | 1.74E-08 | 1.29E-07 |
| Novosphingobium OTU240 | -2.342805088 | 3.588282165 | 5.758465438 | 2.367669344 | 2.278916 | 4.44E-09 | 5.07E-08 |
| Novosphingobium OTU215 | -1.819580731 | 4.229276893 | 5.986513522 | 2.132293065 | 2.614753 | 2.42E-09 | 4.17E-08 |
| Novosphingobium OTU157 | -2.396781138 | 4.134180013 | 6.515178001 | 2.370155639 | 2.657644 | 2.68E-09 | 4.21E-08 |
| Novosphingobium OTU169 | -2.156690462 | 4.081170065 | 6.097550039 | 2.238534581 | 2.668172 | 2.53E-09 | 4.18E-08 |
| Novosphingobium OTU83 | -1.321587453 | 4.369509844 | 5.616995435 | 1.659165327 | 3.244584 | 2.94E-09 | 4.26E-08 |
| Novosphingobium OTU375 | -1.931349496 | 5.143752148 | 7.060358597 | 2.076484466 | 3.361038 | 9.39E-10 | 3.81E-08 |
| Acinetobacter OTU72 | -2.93585972 | 4.432250387 | 6.881197044 | 4.835744272 | 1.351669 | 1.03E-07 | 5.87E-07 |
| Acinetobacter OTU40 | -3.461862135 | 4.951093651 | 7.756404591 | 4.893773589 | 1.507054 | 3.64E-08 | 2.34E-07 |
| Acinetobacter OTU73 | -2.950592986 | 4.746803007 | 7.512493221 | 4.363050345 | 1.643811 | 5.64E-09 | 5.94E-08 |
| Acinetobacter OTU221 | -2.385553745 | 4.499388015 | 6.739103522 | 4.072187807 | 1.653369 | 1.22E-08 | 9.73E-08 |
| Acinetobacter OTU79 | -3.158802527 | 4.425546289 | 7.410077972 | 4.217006591 | 1.655563 | 1.15E-08 | 9.36E-08 |
| Acinetobacter OTU121 | -2.885437473 | 4.550241442 | 7.379474732 | 4.363932011 | 1.661667 | 1.06E-08 | 8.85E-08 |
| Acinetobacter OTU150 | -2.125481821 | 4.644466306 | 6.581066853 | 3.705281722 | 1.675355 | 8.13E-09 | 7.49E-08 |
| Acinetobacter OTU320 | -1.628575886 | 4.331744325 | 5.991095414 | 3.397985026 | 1.68685 | 1.74E-08 | 1.28E-07 |
| Acinetobacter OTU253 | -2.02185355 | 4.815642984 | 6.674434688 | 3.629832366 | 1.750039 | 9.6E-09 | 8.33E-08 |
| Streptomyces OTU272 | -4.499869363 | 0.711030995 | 5.19775935 | 4.168733295 | 1.076928 | 1.22E-05 | 4.03E-05 |
| Streptomyces OTU181 | -4.618667174 | 1.018315572 | 5.64686768 | 3.803709313 | 1.362325 | 5.66E-07 | 2.79E-06 |
| Streptomyces OTU81 | -2.684653719 | 2.467918491 | 5.286069099 | 2.680634115 | 1.842023 | 4.85E-08 | 3.01E-07 |
| Bacillus OTU246 | -5.439417374 | -0.788535003 | 4.82658651 | 5.155463133 | 0.865105 | 0.000187 | 0.000428 |
| Bacillus OTU147 | -5.256484514 | -0.501040755 | 4.862098523 | 4.010228517 | 1.125316 | 2.66E-05 | 7.07E-05 |
| Ottowia OTU543 | -0.066140793 | 1.488731981 | 1.496378453 | 1.812983928 | 0.761587 | 0.000585 | 0.001297 |
| Fictibacillus OTU56 | -5.380451093 | -0.58200133 | 4.849250247 | 4.85635546 | 0.940974 | 0.000187 | 0.000399 |
| Polynucleobacter OTU552 | -0.589232159 | 2.103804142 | 2.605883304 | 1.52811334 | 1.677121 | 2.69E-08 | 1.79E-07 |
| Sphingomonas OTU1456 | -1.324969585 | 4.661014434 | 5.962559284 | 1.802964821 | 3.318096 | 1.03E-09 | 3.81E-08 |
| Luteimonas OTU954 | -1.635040898 | 4.201259616 | 5.807132044 | 2.306435843 | 2.421074 | 1.66E-09 | 4.06E-08 |

**Supplementary Table 8-2: The ALDEX2 output results of the 2 identified low abundant prokaryotic OTUs in Wuhan-May-2020 vs Others**.

| **OTU** | **rab.win.Others** | **rab.win.Wuhan** | **diff.btw** | **diff.win** | **effect** | **wi.ep** | **wi.eBH** |
| --- | --- | --- | --- | --- | --- | --- | --- |
| Candidatus Methylopumilus OTU74 | -0.003967757 | -1.869055249 | -1.81121415 | 2.376016689 | -0.67315 | 0.006276 | 0.008816 |
| Limnohabitans OTU31 | 0.264597024 | -1.680360939 | -2.15570131 | 3.108765503 | -0.63392 | 0.003749 | 0.005877 |

**Supplementary Table 9-1: The ALDEX2 output results of the 26 identified highly abundant microeukaryotic OTUs in Wuhan-May-2020 vs Others.**

| **OTU** | **rab.win.Others** | **rab.win.Wuhan** | **diff.btw** | **diff.win** | **effect** | **wi.ep** | **wi.eBH** |
| --- | --- | --- | --- | --- | --- | --- | --- |
| g__Centropages OTU290 | 0.500742355 | 4.202839286 | 3.34830433 | 3.855711523 | 0.793084476 | 0.000366808 | 0.002906631 |
| g__Choricystis OTU614 | -0.230918859 | 2.920202354 | 3.153876466 | 3.170137958 | 0.850505608 | 5.72E-05 | 0.000688886 |
| g__Choricystis OTU893 | 1.673343178 | 3.550800706 | 2.101843387 | 2.467919373 | 0.746215968 | 0.000207136 | 0.001607324 |
| g__Cyclotella OTU472 | 1.627308947 | 3.921202723 | 2.372712247 | 2.775243021 | 0.722361941 | 0.000187441 | 0.001627522 |
| g__Cyclotella OTU9 | 3.17398306 | 4.285842638 | 1.196848591 | 1.680367198 | 0.614141851 | 0.002158746 | 0.009659514 |
| g__Ettlia OTU917 | 0.102771151 | 2.837584392 | 2.678614868 | 3.05879803 | 0.821282621 | 0.000175738 | 0.001536986 |
| g__Mychonastes OTU138 | 0.322785908 | 2.637358603 | 2.337269721 | 3.032843775 | 0.71061338 | 0.001048318 | 0.005431372 |
| g__Mychonastes OTU187 | 1.166911422 | 3.720249304 | 2.510292207 | 2.603757425 | 0.881100914 | 2.97E-05 | 0.00039236 |
| g__Mychonastes OTU423 | -0.487302344 | 2.309015827 | 2.612981594 | 3.853632557 | 0.631356498 | 0.001847791 | 0.008454519 |
| g__Mychonastes OTU529 | -0.591975038 | 1.915630867 | 2.403251425 | 3.231192713 | 0.68264245 | 0.001794012 | 0.00779344 |
| g__Mychonastes OTU705 | -0.23493602 | 2.491785894 | 2.694433465 | 3.749318447 | 0.655822943 | 0.001619419 | 0.007072391 |
| g__Mychonastes OTU743 | -0.501090367 | 2.668360363 | 2.973085849 | 3.674306467 | 0.752886134 | 0.000621243 | 0.003453804 |
| g__Sinocalanus OTU200 | 0.935852942 | 4.346405952 | 3.314950684 | 3.543197846 | 0.814465337 | 0.00019717 | 0.001841053 |
| g__Sinocalanus OTU280 | 1.592220302 | 4.968900332 | 3.03383338 | 3.910562723 | 0.70641039 | 0.000175419 | 0.001795248 |
| g__Sinocalanus OTU370 | 1.294386387 | 4.710090946 | 3.300507399 | 3.932185129 | 0.756989669 | 0.000204403 | 0.002000249 |
| g__Sinocalanus OTU466 | 1.809027391 | 5.082712333 | 2.936212065 | 4.420055891 | 0.610371531 | 0.000828439 | 0.005735661 |
| g__Sinocalanus OTU551 | 0.872876419 | 3.769582195 | 2.895900697 | 3.869216124 | 0.671741567 | 0.001246578 | 0.006843403 |
| g__Sinocalanus OTU554 | 0.704532413 | 3.279365063 | 2.728015949 | 3.702583734 | 0.649808854 | 0.001683736 | 0.008393184 |
| g__Sinocalanus OTU634 | 1.765743354 | 4.27096542 | 2.457360224 | 3.744433965 | 0.588548212 | 0.001933445 | 0.009939273 |
| g__Skeletonema OTU19 | -0.127878437 | 2.381686509 | 2.407756763 | 3.407922321 | 0.636438785 | 0.001485973 | 0.007256398 |
| g__Skeletonema OTU514 | 0.65707361 | 3.082242761 | 2.384955662 | 3.302549945 | 0.590024233 | 0.00117011 | 0.006741995 |
| g__Skeletonema OTU843 | -0.00236445 | 3.07102808 | 2.972548634 | 3.20558923 | 0.853355886 | 5.69E-05 | 0.000690933 |
| g__Skeletonema OTU844 | 0.121218805 | 2.868467488 | 2.605634888 | 3.291130819 | 0.714888332 | 0.000417538 | 0.003055003 |
| g__Skeletonema OTU99 | 0.12407269 | 3.539885035 | 3.124905658 | 3.547270528 | 0.765506409 | 0.00031199 | 0.002479314 |
| g__Stentor OTU409 | -0.520021336 | 2.75374631 | 3.073129664 | 3.769909326 | 0.722084829 | 0.000988006 | 0.004660533 |
| g__Thalassiosira OTU467 | 0.371226887 | 2.594705365 | 2.352868113 | 3.000234799 | 0.649740344 | 0.002049742 | 0.00787384 |

**Supplementary Table 9-2: The ALDEX2 output results of the 30 identified low abundant microeukaryotic OTUs in Wuhan-May-2020 vs Others.**

| **OTU** | **rab.win.Others** | **rab.win.Wuhan** | **diff.btw** | **diff.win** | **effect** | **wi.ep** | **wi.eBH** |
| --- | --- | --- | --- | --- | --- | --- | --- |
| g__Cryptomonas OTU127 | 4.40332795 | 0.282848425 | -4.2401319 | 2.536794633 | -1.61024796 | 1.58E-08 | 6.41E-06 |
| g__Cryptomonas OTU13 | 3.311783404 | -0.569536052 | -3.7063228 | 3.362234942 | -1.04163574 | 4.85E-06 | 0.000112414 |
| g__Cryptomonas OTU240 | 3.422293476 | -0.588195762 | -3.90304033 | 3.461408099 | -1.03521337 | 5.49E-06 | 0.000125882 |
| g__Cryptomonas OTU255 | 3.942325184 | -0.080627181 | -3.88489899 | 3.005614613 | -1.24686847 | 2.45E-07 | 1.60E-05 |
| g__Cryptomonas OTU259 | 2.307679288 | -0.707751693 | -2.88739525 | 3.575179725 | -0.72713684 | 0.001108076 | 0.005385717 |
| g__Cryptomonas OTU264 | 2.187212629 | -0.745938044 | -2.80456244 | 3.74300689 | -0.69010069 | 0.001304314 | 0.00604934 |
| g__Cryptomonas OTU320 | 2.75130284 | -0.524652066 | -3.07402772 | 3.494240627 | -0.79388487 | 0.000326477 | 0.002159783 |
| g__Cryptomonas OTU454 | 4.099594113 | -0.425587955 | -4.48934508 | 2.861092195 | -1.38867417 | 1.38E-07 | 1.24E-05 |
| g__Cryptomonas OTU51 | 3.788956183 | -0.809009146 | -4.4531494 | 3.080247167 | -1.35945809 | 3.15E-07 | 1.84E-05 |
| g__Cryptomonas OTU568 | 2.943117007 | -0.793670201 | -3.66670088 | 3.504466949 | -0.9438605 | 0.00010194 | 0.000914365 |
| g__Cryptomonas OTU570 | 2.459777118 | -0.652685117 | -3.09259794 | 3.54406271 | -0.78899953 | 0.000374138 | 0.002407714 |
| g__Cryptomonas OTU578 | 2.038124685 | -0.839694063 | -2.69442172 | 3.721829458 | -0.63314865 | 0.002188528 | 0.008523594 |
| g__Cryptomonas OTU580 | 2.943942021 | -0.708501324 | -3.47191444 | 3.424098117 | -0.86736272 | 8.74E-05 | 0.000802997 |
| g__Cryptomonas OTU620 | 3.317625965 | -0.652248254 | -3.85961881 | 3.304156678 | -1.06741586 | 6.47E-06 | 0.000153331 |
| g__Cryptomonas OTU665 | 3.370704313 | -0.208105929 | -3.50809484 | 3.12613604 | -1.01072013 | 1.54E-05 | 0.000215861 |
| g__Cryptomonas OTU686 | 3.211121184 | -0.794684468 | -3.88097151 | 3.327276747 | -1.05812105 | 8.87E-06 | 0.000153474 |
| g__Cryptomonas OTU70 | 3.773245353 | -0.888051655 | -4.39694953 | 3.31481539 | -1.2502216 | 7.86E-07 | 3.33E-05 |
| g__Cryptomonas OTU745 | 3.012541245 | -0.674972588 | -3.51762942 | 3.258401522 | -0.98207028 | 2.24E-05 | 0.000341432 |
| g__Cryptomonas OTU75 | 3.549244056 | -0.599210637 | -4.07817808 | 3.11750028 | -1.19664278 | 2.12E-06 | 5.95E-05 |
| g__Cryptomonas OTU777 | 3.237653147 | 0.878965532 | -2.27918805 | 2.863699652 | -0.71458804 | 0.000498224 | 0.003071111 |
| g__Cryptomonas OTU85 | 3.356266339 | -0.735670856 | -3.90479908 | 3.269887922 | -1.12718298 | 1.79E-06 | 6.03E-05 |
| g__Cryptomonas OTU857 | 2.239763489 | -0.862049456 | -2.8783898 | 3.642104348 | -0.7089697 | 0.001358187 | 0.005973221 |
| g__Cryptomonas OTU870 | 2.958831786 | -0.818057194 | -3.58286395 | 3.355167836 | -0.96025626 | 3.65E-05 | 0.000398116 |
| g__Cryptomonas OTU888 | 3.508914352 | 0.150494874 | -3.29116455 | 2.84316795 | -1.06006944 | 5.77E-06 | 0.000109662 |
| g__Cryptomonas OTU928 | 4.64551869 | 2.40785614 | -2.21899846 | 1.82369064 | -1.09524571 | 1.32E-06 | 4.76E-05 |
| g__Cryptomonas OTU955 | 3.642371181 | -0.706880201 | -4.18218064 | 3.275915322 | -1.23556864 | 1.26E-06 | 3.90E-05 |
| g__Cryptomonas OTU96 | 4.090245642 | -0.582315943 | -4.68586638 | 3.124738911 | -1.40522706 | 1.21E-07 | 1.20E-05 |
| g__Cryptomonas OTU982 | 2.383458142 | -0.744880751 | -2.90228556 | 3.807666322 | -0.71517802 | 0.000754974 | 0.003953728 |
| g__Parastrombidinopsis OTU122 | 2.679510937 | -0.620198435 | -3.07965308 | 3.72929712 | -0.77232183 | 0.000272941 | 0.002067436 |
| g__Salpingella OTU773 | 2.833882735 | -0.419376795 | -3.02894074 | 3.478734932 | -0.7414977 | 0.000207495 | 0.001735126 |

**Supplementary Table 10: The 17 identified significant different KEGG pathways in Wuhan-May-2020 vs Others.**

| **ID** | **Description** | **setSize** | **enrichmentScore** | ***p*-value** | ***p*.adjust** | **q-value** |
| --- | --- | --- | --- | --- | --- | --- |
| ko01053 | Biosynthesis of siderophore group nonribosomal peptides | 18 | 0.748878933 | 5.24E-05 | 0.001135 | 0.000907 |
| ko00571 | Lipoarabinomannan (LAM) biosynthesis | 10 | 0.734319864 | 0.003259 | 0.037381 | 0.029865 |
| ko04112 | Cell cycle - Caulobacter | 25 | 0.657263876 | 0.000461 | 0.00817 | 0.006527 |
| ko02026 | Biofilm formation - Escherichia coli | 27 | 0.583792866 | 0.002691 | 0.032797 | 0.026203 |
| ko02024 | Quorum sensing | 87 | 0.550946446 | 3.29E-07 | 2.14E-05 | 1.71E-05 |
| ko02025 | Biofilm formation - Pseudomonas aeruginosa | 44 | 0.528837724 | 0.002368 | 0.030789 | 0.024598 |
| ko02020 | Two-component system | 187 | 0.46214023 | 1.55E-07 | 1.51E-05 | 1.21E-05 |
| ko02010 | ABC transporters | 176 | 0.433782856 | 1.68E-05 | 0.000468 | 0.000374 |
| ko01250 | Biosynthesis of nucleotide sugars | 89 | -0.400373238 | 0.000281 | 0.005478 | 0.004376 |
| ko03010 | Ribosome | 74 | -0.478984365 | 4.52E-06 | 0.000147 | 0.000117 |
| ko00680 | Methane metabolism | 74 | -0.492918118 | 1.5E-06 | 7.32E-05 | 5.85E-05 |
| ko03440 | Homologous recombination | 26 | -0.549783909 | 0.002103 | 0.029298 | 0.023407 |
| ko04010 | MAPK signaling pathway | 18 | -0.635256236 | 0.000984 | 0.015986 | 0.012771 |
| ko05171 | Coronavirus disease - COVID-19 | 26 | -0.637134051 | 5.17E-05 | 0.001135 | 0.000907 |
| ko03030 | DNA replication | 32 | -0.641378059 | 3.51E-06 | 0.000137 | 0.000109 |
| ko05225 | Hepatocellular carcinoma | 10 | -0.743054403 | 0.001315 | 0.019721 | 0.015755 |
| ko03020 | RNA polymerase | 23 | -0.767321355 | 1.18E-07 | 1.51E-05 | 1.21E-05 |

The dashed line separated the upregulated and downregulated pathways in Wuhan May 2020

**Supplementary Table 11: The significance test of prokaryotic composition change and recovery by pair-comparisons of KL divergence.**

| **Pair-comparisons** | **estimate** | **SE** | **df** | **t.ratio** | ***p*-value** |
| --- | --- | --- | --- | --- | --- |
| Wuhan_May-Jun.2020 - Yueyang_May-Jun.2020 | 1.9813 | 0.188 | 152 | 10.55 | <.0001 |
| Jiujiang_May-Jun.2020 - Yueyang_May-Jun.2020 | 4.4106 | 0.188 | 152 | 23.49 | <.0001 |
| Nanjing_May-Jun.2020 - Yueyang_May-Jun.2020 | 1.7762 | 0.18 | 152 | 9.88 | <.0001 |
| (Wuhan_May-Jun.2020 - Yueyang_May-Jun.2020) - (Wuhan_Jan.2020 - Yueyang_Jan.2020) | 1.9898 | 0.181 | 77 | 11.01 | 0 |
| (Jiujiang_May-Jun.2020 - Yueyang_May-Jun.2020) - (Jiujiang_Jan.2020 - Yueyang_Jan.2020) | 4.4041 | 0.301 | 77 | 14.62 | 0 |
| (Nanjing_May-Jun.2020 - Yueyang_May-Jun.2020) - (Nanjing_Jan.2020 - Yueyang_Jan.2020) | 0.4456 | 0.254 | 152 | 1.75 | 1 |
| (Wuhan_Oct.2020 - Yueyang_Oct.2020) - (Wuhan_Jan.2020 - Yueyang_Jan.2020) | 0.5449 | 0.181 | 77 | 3.02 | 0.3692 |
| (Jiujiang_Oct.2021 - Yueyang_Oct.2021) - (Jiujiang_Jan.2020 - Yueyang_Jan.2020) | -0.1613 | 0.298 | 77 | -0.54 | 1 |
| (Nanjing_Oct.2020 - Yueyang_Oct.2020) - (Nanjing_Jan.2020 - Yueyang_Jan.2020) | -0.9410 | 0.254 | 152 | -3.70 | 0.4295 |
| (Wuhan_May.2021 - Yueyang_May.2021) - (Wuhan_Jan.2020 - Yueyang_Jan.2020) | 0.1724 | 0.181 | 77 | 0.95 | 1 |
| (Jiujiang_May.2021 - Yueyang_May.2021) - (Jiujiang_Jan.2020 - Yueyang_Jan.2020) | -0.0931 | 0.298 | 77 | -0.31 | 1 |
| (Nanjing_May.2021 - Yueyang_May.2021) - (Nanjing_Jan.2020 - Yueyang_Jan.2020) | -0.8197 | 0.254 | 152 | -3.22 | 0.8202 |

**Supplementary Table 12: The significance test of microeukaryotic composition change and recovery by pair-comparisons of KL divergence.**

| **Pair-comparisons** | **estimate** | **SE** | **df** | **t.ratio** | ***p*-value** |
| --- | --- | --- | --- | --- | --- |
| Wuhan_May-Jun.2020 - Yueyang_May-Jun.2020 | 1.8130 | 0.135 | 70 | 13.469 | <.0001 |
| Jiujiang_May-Jun.2020 - Yueyang_May-Jun.2020 | 1.4153 | 0.298 | 62 | 4.745 | 0.0003 |
| Nanjing_May-Jun.2020 - Yueyang_May-Jun.2020 | 0.3392 | 0.137 | 68 | 2.468 | 0.2271 |
| (Wuhan_May-Jun.2020 - Yueyang_May-Jun.2020) - (Wuhan_Jan.2020 - Yueyang_Jan.2020) | 0.9713 | 0.218 | 70 | 4.4496 | 0.0084 |
| (Jiujiang_May-Jun.2020 - Yueyang_May-Jun.2020) - (Jiujiang_Jan.2020 - Yueyang_Jan.2020) | 1.1568 | 0.44 | 62 | 2.6278 | 0.6495 |
| (Nanjing_May-Jun.2020 - Yueyang_May-Jun.2020) - (Nanjing_Jan.2020 - Yueyang_Jan.2020) | -0.5255 | 0.217 | 68 | -2.425 | 0.7864 |
| (Wuhan_Oct.2020 - Yueyang_Oct.2020) - (Wuhan_Jan.2020 - Yueyang_Jan.2020) | -0.5514 | 0.218 | 70 | -2.526 | 0.7216 |
| (Jiujiang_Oct.2021 - Yueyang_Oct.2021) - (Jiujiang_Jan.2020 - Yueyang_Jan.2020) | 0.4979 | 0.407 | 62 | 1.2222 | 1 |
| (Nanjing_Oct.2020 - Yueyang_Oct.2020) - (Nanjing_Jan.2020 - Yueyang_Jan.2020) | -0.4605 | 0.233 | 68 | -1.974 | 0.9661 |
| (Wuhan_May.2021 - Yueyang_May.2021) - (Wuhan_Jan.2020 - Yueyang_Jan.2020) | -0.6367 | 0.216 | 70 | -2.944 | 0.4199 |
| (Jiujiang_May.2021 - Yueyang_May.2021) - (Jiujiang_Jan.2020 - Yueyang_Jan.2020) | 0.2471 | 0.401 | 62 | 0.6169 | 1 |
| (Nanjing_May.2021 - Yueyang_May.2021) - (Nanjing_Jan.2020 - Yueyang_Jan.2020) | -0.1215 | 0.215 | 68 | -0.566 | 1 |

**Supplementary Table 13: The significance test of community function change and recovery by pair-comparisons of KL divergence.**

| **Pair-comparisons** | **estimate** | **SE** | **df** | **t.ratio** | ***p*-value** |
| --- | --- | --- | --- | --- | --- |
| Wuhan_May-Jun.2020 - Yueyang_May-Jun.2020 | 0.1574 | 0.009 | 81 | 17.564 | <.0001 |
| Jiujiang_May-Jun.2020 - Yueyang_May-Jun.2020 | 0.3454 | 0.03 | 77 | 11.411 | <.0001 |
| Nanjing_May-Jun.2020 - Yueyang_May-Jun.2020 | 0.0582 | 0.01 | 87 | 5.97 | <.0001 |
| (Wuhan_May-Jun.2020 - Yueyang_May-Jun.2020) - (Wuhan_Jan.2020 - Yueyang_Jan.2020) | 0.1582 | 0.013 | 81 | 11.783 | 3E-11 |
| (Jiujiang_May-Jun.2020 - Yueyang_May-Jun.2020) - (Jiujiang_Jan.2020 - Yueyang_Jan.2020) | 0.3420 | 0.043 | 77 | 7.8746 | 6E-09 |
| (Nanjing_May-Jun.2020 - Yueyang_May-Jun.2020) - (Nanjing_Jan.2020 - Yueyang_Jan.2020) | 0.0408 | 0.014 | 87 | 2.8713 | 0.4678 |
| (Wuhan_Oct.2020 - Yueyang_Oct.2020) - (Wuhan_Jan.2020 - Yueyang_Jan.2020) | 0.0056 | 0.014 | 81 | 0.4042 | 1 |
| (Jiujiang_Oct.2021 - Yueyang_Oct.2021) - (Jiujiang_Jan.2020 - Yueyang_Jan.2020) | -0.0002 | 0.043 | 77 | -0.004 | 1 |
| (Nanjing_Oct.2020 - Yueyang_Oct.2020) - (Nanjing_Jan.2020 - Yueyang_Jan.2020) | -0.0228 | 0.014 | 87 | -1.591 | 0.9982 |
| (Wuhan_May.2021 - Yueyang_May.2021) - (Wuhan_Jan.2020 - Yueyang_Jan.2020) | -0.0095 | 0.014 | 81 | -0.692 | 1 |
| (Jiujiang_May.2021 - Yueyang_May.2021) - (Jiujiang_Jan.2020 - Yueyang_Jan.2020) | -0.0137 | 0.043 | 77 | -0.32 | 1 |
| (Nanjing_May.2021 - Yueyang_May.2021) - (Nanjing_Jan.2020 - Yueyang_Jan.2020) | -0.0018 | 0.014 | 87 | -0.127 | 1 |

**Supplementary Table 14: MAGs information about carrying both ARGs and MGEs.**

| **MAGs Type** | **MAGs number** |
| --- | --- |
| MAG (contain ARGs) | 653 |
| MAG (contain MGEs) | 4461 |
| MAG (contain ARGs&MGEs) | 648 |

The detail information of ARGs/MGEs count in each MAG (contain ARGs&MGEs) is in Supplementary Excel file.

**Supplementary Table 15: Relative abundance of pathogenetic and antimicrobial resistant bacteria (PARB), antibiotic resistant bacteria (ARB), and pathogens.**

| **PARB** | **Yueyang** | **Wuhan** | **Jiujiang** | **Nanjing** |
| --- | --- | --- | --- | --- |
| Jan. 2020 | 0.3% | 0.3% | 0.4% | 0.6% |
| May-Jun. 2020 | 0.1% | 1.6% | 1.2% | 1.1% |
| Oct. 2020 | 0.1% | 0.1% | 0.0% | 0.1% |
| May 2021 | 0.2% | 0.2% | 0.5% | 1.0% |

| **ARB** | **Yueyang** | **Wuhan** | **Jiujiang** | **Nanjing** |
| --- | --- | --- | --- | --- |
| Jan. 2020 | 6.2% | 6.9% | 5.7% | 4.3% |
| May-Jun. 2020 | 2.8% | 7.0% | 10.2% | 6.1% |
| Oct. 2020 | 3.5% | 2.5% | 2.1% | 2.2% |
| May 2021 | 7.1% | 5.3% | 7.5% | 4.6% |

| **Pathogens** | **Yueyang** | **Wuhan** | **Jiujiang** | **Nanjing** |
| --- | --- | --- | --- | --- |
| Jan. 2020 | 0.6% | 0.7% | 0.6% | 0.6% |
| May-Jun. 2020 | 0.5% | 1.1% | 0.4% | 1.0% |
| Oct. 2020 | 0.5% | 0.5% | 0.6% | 0.8% |
| May 2021 | 0.8% | 0.7% | 1.6% | 1.0% |

**PARB**: bacterial contains both ARG(s) and VFG(s).

**ARB**: bacterial contains ARG(s) without VFG(s).

**Pathogens**: bacterial contains VFG(s) without ARG(s).

**Supplementary Table 16: Mean cell density of pathogenetic and antimicrobial resistant bacteria (cells/ml).**

| **Region** | **Yueyang** | **Wuhan** | **Jiujiang** | **Nanjing** |
| --- | --- | --- | --- | --- |
| Jan. 2020 | 8.38E+01 | 7.12E+01 | 1.76E+02 | 2.21E+02 |
| May-Jun. 2020 | 5.67E+01 | 1.81E+02 | 8.72E+02 | 1.75E+03 |
| Oct. 2020 | 6.13E+02 | 1.30E+03 | 4.88E+02 | 1.12E+03 |
| May 2021 | 4.78E+03 | 7.06E+02 | 4.34E+03 | 1.04E+04 |

The cell density of PARB was the product of PARB relative abundance and total cell density (flow cytometry). The low total cell density in Wuhan-May-2020 (Supplementary Table 3) explained why cell density of PARB was low inWuhan-May-2020.

**Supplementary Table 17: The significance test of PARB community change and recovery by pair-comparisons of KL divergence.**

| **Pair-comparisons** | **estimate** | **SE** | **df** | **t.ratio** | ***p*-value** |
| --- | --- | --- | --- | --- | --- |
| Wuhan_May-Jun.2020 - Yueyang_May-Jun.2020 | 1.98 | 0.188 | 55.5 | 10.54 | <.0001 |
| Jinjiang_May-Jun.2020 - Yueyang_May-Jun.2020 | 4.41 | 0.189 | 51 | 23.47 | <.0001 |
| Nanjing May-Jun.2020 - Yueyang_May-Jun.2020 | 1.78 | 0.18 | 50.8 | 9.87 | <.0001 |
| (Wuhan_May-Jun.2020 - Yueyang_May-Jun.2020) - (Wuhan_Jan.2020 - Yueyang_Jan.2020) | 1.90 | 0.266 | 57.9 | 7.16 | <.0001 |
| (Jiujiang_May-Jun.2020 - Yueyang_May-Jun.2020) - (Jiujiang_Jan.2020 - Yueyang_Jan.2020) | 4.32 | 0.266 | 49.0 | 16.25 | <.0001 |
| (Nanjing_May-Jun.2020 - Yueyang_May-Jun.2020) - (Nanjing_Jan.2020 - Yueyang_Jan.2020) | 2.04 | 0.452 | 87.0 | 4.51 | <0.01 |
| (Wuhan_Oct.2020 - Yueyang_Oct.2020) - (Wuhan_Jan.2020 - Yueyang_Jan.2020) | 0.44 | 0.266 | 60.4 | 1.66 | 1 |
| (Jiujiang_Oct.2020 - Yueyang_Oct.2020) - (Jiujiang_Jan.2020 - Yueyang_Jan.2020) | -0.26 | 0.266 | 53.2 | -0.98 | 1 |
| (Nanjing_Oct.2020 - Yueyang_Oct.2020) - (Nanjing_Jan.2020 - Yueyang_Jan.2020) | 0.30 | 0.455 | 87.0 | 0.66 | 1 |
| (Wuhan_May.2021 - Yueyang_May.2021) - (Wuhan_Jan.2020 - Yueyang_Jan.2020) | 0.09 | 0.266 | 53.2 | 0.33 | 1 |
| (Jiujiang_May.2021 - Yueyang_May.2021) - (Jiujiang_Jan.2020 - Yueyang_Jan.2020) | -0.17 | 0.266 | 51.0 | -0.65 | 1 |
| (Nanjing_May.2021 - Yueyang_May.2021) - (Nanjing_Jan.2020 - Yueyang_Jan.2020) | 1.56 | 0.459 | 87.0 | 3.41 | 0.16 |

**Supplementary Table 18: River flow data.**

| **Water flow (m3/s)** | **Wuhan** | **Jiujiang** | **Nanjing** |
| --- | --- | --- | --- |
| Jan. 2020 | 12399.19 | 12821.09 | 14762.54 |
| May-June. 2020 | 24399.43 | 25059.18 | 30687.35 |
| Oct. 2020 | 35401.73 | 37447.73 | 45549.58 |
| May. 2021 | 34098.72 | 34636.35 | 40621.27 |

Data source: Changjiang water resources commission of the ministry of water resources http://www.cjw.gov.cn/zwzc/bmgb/ (in Chinese)

The data in Yueyang was missing.

**Supplementary Table 19: General descriptions of the metagenomic dataset in each sample.**

| **Sample name** | **Clean Reads** | **Clean Base** | **Read Length** | **Q20(%)** | **GC(%)** |
| --- | --- | --- | --- | --- | --- |
| JJ1_June_2020 | 82022422 | 12303363300 | 150;150 | 96.52 | 48.29 |
| JJ2_June_2020 | 67118636 | 10067795400 | 150;150 | 94.09 | 58.85 |
| JJ3_June_2020 | 77295292 | 11594293800 | 150;150 | 92.96 | 64.86 |
| JJ4_June_2020 | 72572672 | 10885900800 | 150;150 | 93.38 | 62.96 |
| JJ5_June_2020 | 64610524 | 9691578600 | 150;150 | 93.45 | 60.32 |
| NJ1_June_2020 | 76405940 | 11460891000 | 150;150 | 94.85 | 56.71 |
| NJ2_June_2020 | 73742640 | 11061396000 | 150;150 | 93.98 | 60.04 |
| NJ3_June_2020 | 90563874 | 13584581100 | 150;150 | 94.54 | 57.02 |
| NJ4_June_2020 | 76046276 | 11406941400 | 150;150 | 95 | 54.34 |
| NJ5_June_2020 | 75471588 | 11320738200 | 150;150 | 94.01 | 58.87 |
| NJ6_June_2020 | 77644728 | 11646709200 | 150;150 | 94.31 | 57.73 |
| NJ7_June_2020 | 80923738 | 12138560700 | 150;150 | 94.6 | 56.43 |
| NJ8_June_2020 | 86668402 | 13000260300 | 150;150 | 94.84 | 55.11 |
| NJ9_June_2020 | 82341506 | 12351225900 | 150;150 | 95.81 | 57.03 |
| NJ10_June_2020 | 96455708 | 14468356200 | 150;150 | 94.84 | 56.75 |
| NJ11_June_2020 | 80522480 | 12078372000 | 150;150 | 95.03 | 58.16 |
| NJ12_June_2020 | 63597944 | 9539691600 | 150;150 | 94.03 | 60.43 |
| JJ7_June_2020 | 92897054 | 13934558100 | 150;150 | 94.99 | 53.7 |
| JJ8_June_2020 | 87273368 | 13091005200 | 150;150 | 93.91 | 59.81 |
| JJ9_June_2020 | 72179108 | 10826866200 | 150;150 | 92.77 | 66.3 |
| JJ10_June_2020 | 71787290 | 10768093500 | 150;150 | 93.62 | 61.07 |
| JJ11_June_2020 | 86068820 | 12910323000 | 150;150 | 94.36 | 56.63 |
| NJ13_June_2020 | 95865692 | 14379853800 | 150;150 | 94.49 | 56.37 |
| NJ14_June_2020 | 64138594 | 9620789100 | 150;150 | 93.95 | 57.81 |
| YY1_June_2020 | 90036774 | 13505516100 | 150;150 | 95.12 | 50.07 |
| YY2_June_2020 | 87537170 | 13130575500 | 150;150 | 95.19 | 48.4 |
| YY3_June_2020 | 76696614 | 11504492100 | 150;150 | 94.63 | 50.89 |
| YY4_June_2020 | 80633712 | 12095056800 | 150;150 | 95.19 | 50.57 |
| YY5_June_2020 | 69489552 | 10423432800 | 150;150 | 94.81 | 51.18 |
| YY6_June_2020 | 76644378 | 11496656700 | 150;150 | 94.69 | 51.09 |
| YY7_June_2020 | 74199076 | 11129861400 | 150;150 | 94.53 | 50.12 |
| YY8_June_2020 | 90577628 | 13586644200 | 150;150 | 95.22 | 50.14 |
| YY9_June_2020 | 88587162 | 13288074300 | 150;150 | 95.02 | 51.48 |
| YY10_June_2020 | 83321682 | 12498252300 | 150;150 | 94.72 | 50.8 |
| YY11_June_2020 | 71889952 | 10783492800 | 150;150 | 94.38 | 51.18 |
| NJ2_January_2020 | 84076714 | 12611507100 | 150;150 | 93.66 | 48.43 |
| NJ4_January_2020 | 87295952 | 13094392800 | 150;150 | 93.67 | 49.11 |
| NJ10_January_2020 | 104357344 | 15653601600 | 150;150 | 94.27 | 50.73 |
| NJ11_January_2020 | 70708244 | 10606236600 | 150;150 | 93.42 | 47.05 |
| NJ1_January_2020 | 88166402 | 13224960300 | 150;150 | 94.12 | 46.15 |
| NJ3_January_2020 | 86443994 | 12966599100 | 150;150 | 94.17 | 48.33 |
| NJ5_January_2020 | 75946788 | 11392018200 | 150;150 | 93.47 | 47.36 |
| NJ6_January_2020 | 83511518 | 12526727700 | 150;150 | 94.01 | 46.9 |
| NJ7_January_2020 | 77471374 | 11620706100 | 150;150 | 93.44 | 47.25 |
| NJ8_January_2020 | 73673578 | 11051036700 | 150;150 | 93.97 | 48.67 |
| NJ9_January_2020 | 78211974 | 11731796100 | 150;150 | 94.24 | 46.99 |
| NJ12_January_2020 | 83928042 | 12589206300 | 150;150 | 93.95 | 47.63 |

**Supplementary Table 19** **continued.**

| **Sample name** | **Clean Reads** | **Clean Base** | **Read Length** | **Q20(%)** | **GC(%)** |
| --- | --- | --- | --- | --- | --- |
| NJ13_January_2020 | 68659482 | 10298922300 | 150;150 | 94.11 | 47 |
| NJ14_January_2020 | 80793056 | 12118958400 | 150;150 | 94.26 | 46.98 |
| JJ1_January_2020 | 82364926 | 12354738900 | 150;150 | 96.64 | 47.96 |
| JJ2_January_2020 | 78925684 | 11838852600 | 150;150 | 96.32 | 48.16 |
| JJ3_January_2020 | 82244318 | 12336647700 | 150;150 | 96.57 | 49.24 |
| JJ4_January_2020 | 82297508 | 12344626200 | 150;150 | 96.4 | 48.23 |
| JJ5_January_2020 | 68268676 | 10240301400 | 150;150 | 96.15 | 52.12 |
| JJ6_January_2020 | 82210768 | 12331615200 | 150;150 | 96.8 | 48.87 |
| JJ7_January_2020 | 82209660 | 12331449000 | 150;150 | 96.27 | 47.53 |
| JJ8_January_2020 | 82162818 | 12324422700 | 150;150 | 96.44 | 47.77 |
| JJ9_January_2020 | 82215154 | 12332273100 | 150;150 | 96.38 | 47.27 |
| JJ10_January_2020 | 81586036 | 12237905400 | 150;150 | 96.42 | 47.91 |
| JJ11_January_2020 | 81976030 | 12296404500 | 150;150 | 96.61 | 47.34 |
| YY1_January_2020 | 81659220 | 12248883000 | 150;150 | 97.09 | 48.42 |
| YY2_January_2020 | 82341038 | 12351155700 | 150;150 | 97.01 | 48.3 |
| YY3_January_2020 | 81045468 | 12156820200 | 150;150 | 96.63 | 50.31 |
| YY4_January_2020 | 82043010 | 12306451500 | 150;150 | 96.73 | 48.64 |
| YY5_January_2020 | 83057768 | 12458665200 | 150;150 | 96.66 | 50.26 |
| YY6_January_2020 | 82537768 | 12380665200 | 150;150 | 97.22 | 49.8 |
| YY7_January_2020 | 81736712 | 12260506800 | 150;150 | 96.86 | 51.8 |
| YY8_January_2020 | 81983302 | 12297495300 | 150;150 | 96.89 | 51.14 |
| YY9_January_2020 | 80999524 | 12149928600 | 150;150 | 97.05 | 49.27 |
| YY10_January_2020 | 81828142 | 12274221300 | 150;150 | 97.25 | 49.78 |
| WH1_January_2020 | 81625026 | 12243753900 | 150;150 | 96.75 | 49.12 |
| WH2_January_2020 | 82210736 | 12331610400 | 150;150 | 96.53 | 49.36 |
| WH3_January_2020 | 82072900 | 12310935000 | 150;150 | 96.8 | 48.63 |
| WH4_January_2020 | 82045012 | 12306751800 | 150;150 | 96.81 | 49.16 |
| WH6_January_2020 | 81055312 | 12158296800 | 150;150 | 96.29 | 48.54 |
| WH7_January_2020 | 71258506 | 10688775900 | 150;150 | 96.36 | 49.67 |
| WH8_January_2020 | 81677636 | 12251645400 | 150;150 | 96.82 | 48.42 |
| WH9_January_2020 | 82677470 | 12401620500 | 150;150 | 96.77 | 48.17 |
| WH10_January_2020 | 81427570 | 12214135500 | 150;150 | 96.49 | 49.22 |
| WH11_January_2020 | 83520466 | 12528069900 | 150;150 | 96.72 | 49.47 |
| H2_January_2020 | 80942354 | 12141353100 | 150;150 | 97.07 | 46.98 |
| WH1_May_2020 | 73174442 | 10976166300 | 150;150 | 95.22 | 57.06 |
| WH2_May_2020 | 64253504 | 9638025600 | 150;150 | 94.91 | 60.36 |
| WH3_May_2020 | 68928866 | 10339329900 | 150;150 | 95.03 | 58.94 |
| WH4_May_2020 | 68642180 | 10296327000 | 150;150 | 95.23 | 56.43 |
| WH5_May_2020 | 68757602 | 10313640300 | 150;150 | 95.01 | 57.52 |
| WH6_May_2020 | 63847068 | 9577060200 | 150;150 | 94.83 | 55.92 |
| WH7_May_2020 | 82972812 | 12445921800 | 150;150 | 95.96 | 54.2 |
| WH8_May_2020 | 82154036 | 12323105400 | 150;150 | 95.18 | 62.3 |
| WH9_May_2020 | 80626278 | 12093941700 | 150;150 | 95.45 | 62.05 |
| WH10_May_2020 | 81426490 | 12213973500 | 150;150 | 95.73 | 59.08 |
| WH11_May_2020 | 81293014 | 12193952100 | 150;150 | 95.45 | 58.65 |
| H1_May_2020 | 81520172 | 12228025800 | 150;150 | 95.43 | 59.08 |
| H2_May_2020 | 72488228 | 10873234200 | 150;150 | 95.52 | 57.47 |

**Supplementary Table 19** **continued.**

| **Sample name** | **Clean Reads** | **Clean Base** | **Read Length** | **Q20(%)** | **GC(%)** |
| --- | --- | --- | --- | --- | --- |
| H3_May_2020 | 80489610 | 12073441500 | 150;150 | 95.86 | 58.21 |
| YY1_October_2020 | 71406760 | 10711014000 | 150;150 | 95.13 | 49.55 |
| YY2_October_2020 | 69134722 | 10370208300 | 150;150 | 94.75 | 47.57 |
| YY3_October_2020 | 71584932 | 10737739800 | 150;150 | 94.44 | 49.16 |
| YY4_October_2020 | 71416524 | 10712478600 | 150;150 | 94.62 | 49.59 |
| YY5_October_2020 | 69334482 | 10400172300 | 150;150 | 94.32 | 48.03 |
| YY6_October_2020 | 71744666 | 10761699900 | 150;150 | 94.71 | 48.86 |
| YY7_October_2020 | 72434948 | 10865242200 | 150;150 | 94.54 | 49.17 |
| YY8_October_2020 | 73476382 | 11021457300 | 150;150 | 94.57 | 48.49 |
| YY9_October_2020 | 73066740 | 10960011000 | 150;150 | 94.42 | 49.17 |
| YY10_October_2020 | 71890164 | 10783524600 | 150;150 | 94.73 | 48.42 |
| YY11_October_2020 | 69913042 | 10486956300 | 150;150 | 94.07 | 48.93 |
| WH1_October_2020 | 72255962 | 10838394300 | 150;150 | 94.78 | 48.89 |
| WH2_October_2020 | 72486890 | 10873033500 | 150;150 | 94.89 | 47.89 |
| WH3_October_2020 | 72142810 | 10821421500 | 150;150 | 94.61 | 48.54 |
| WH4_October_2020 | 70838882 | 10625832300 | 150;150 | 95.05 | 49.78 |
| WH5_October_2020 | 71722782 | 10758417300 | 150;150 | 94.61 | 48.66 |
| WH6_October_2020 | 71540760 | 10731114000 | 150;150 | 94.45 | 49.67 |
| WH7_October_2020 | 72757820 | 10913673000 | 150;150 | 94.85 | 48.99 |
| WH8_October_2020 | 71984338 | 10797650700 | 150;150 | 94.53 | 48.38 |
| WH9_October_2020 | 72409804 | 10861470600 | 150;150 | 94.4 | 48.28 |
| WH10_October_2020 | 60972968 | 9145945200 | 150;150 | 94.34 | 49.43 |
| WH11_October_2020 | 72541460 | 10881219000 | 150;150 | 94.23 | 48.99 |
| JJ1_October_2020 | 71114054 | 10667108100 | 150;150 | 93.3 | 53.46 |
| JJ2_October_2020 | 71115840 | 10667376000 | 150;150 | 93.23 | 48.68 |
| JJ3_October_2020 | 71215382 | 10682307300 | 150;150 | 93.23 | 49.4 |
| JJ4_October_2020 | 65524764 | 9828714600 | 150;150 | 92.96 | 49.45 |
| JJ5_October_2020 | 72336160 | 10850424000 | 150;150 | 93.29 | 49.46 |
| JJ6_October_2020 | 64781816 | 9717272400 | 150;150 | 92.83 | 52.14 |
| JJ7_October_2020 | 72543334 | 10881500100 | 150;150 | 93.69 | 50.32 |
| JJ8_October_2020 | 72667490 | 10900123500 | 150;150 | 93.31 | 49.52 |
| JJ9_October_2020 | 67853382 | 10178007300 | 150;150 | 93.05 | 50.76 |
| JJ10_October_2020 | 71056212 | 10658431800 | 150;150 | 93.37 | 49.7 |
| JJ11_October_2020 | 64871800 | 9730770000 | 150;150 | 93.06 | 48.71 |
| NJ1_October_2020 | 71222954 | 10683443100 | 150;150 | 92.95 | 52.2 |
| NJ2_October_2020 | 65974150 | 9896122500 | 150;150 | 93.2 | 49.08 |
| NJ3_October_2020 | 71010838 | 10651625700 | 150;150 | 93.29 | 49.27 |
| NJ4_October_2020 | 70623582 | 10593537300 | 150;150 | 93.45 | 49.6 |
| NJ5_October_2020 | 71716718 | 10757507700 | 150;150 | 93.53 | 47.83 |
| NJ6_October_2020 | 64456828 | 9668524200 | 150;150 | 92.92 | 49.28 |
| NJ7_October_2020 | 71466836 | 10720025400 | 150;150 | 95.03 | 48.82 |
| NJ8_October_2020 | 71542046 | 10731306900 | 150;150 | 94.68 | 47.99 |
| NJ9_October_2020 | 71441760 | 10716264000 | 150;150 | 94.41 | 48.2 |
| NJ10_October_2020 | 71500306 | 10725045900 | 150;150 | 94.16 | 49.68 |
| NJ12_October_2020 | 71563574 | 10734536100 | 150;150 | 94.2 | 49.41 |
| NJ13_October_2020 | 71584522 | 10737678300 | 150;150 | 94.15 | 48.9 |
| NJ14_October_2020 | 70856110 | 10628416500 | 150;150 | 95.41 | 50.57 |

**Supplementary Table 19** **continued.**

| **Sample name** | **Clean Reads** | **Clean Base** | **Read Length** | **Q20(%)** | **GC(%)** |
| --- | --- | --- | --- | --- | --- |
| YY1_May_2021 | 71540854 | 10731128100 | 150;150 | 96.11 | 54.32 |
| YY2_May_2021 | 71668130 | 10750219500 | 150;150 | 96.09 | 56.04 |
| YY3_May_2021 | 72808866 | 10921329900 | 150;150 | 96.18 | 54.43 |
| YY4_May_2021 | 71336814 | 10700522100 | 150;150 | 96.32 | 53.9 |
| YY5_May_2021 | 71528622 | 10729293300 | 150;150 | 96.09 | 53.51 |
| YY6_May_2021 | 71470088 | 10720513200 | 150;150 | 96.15 | 54.55 |
| YY7_May_2021 | 70762284 | 10614342600 | 150;150 | 96.25 | 53.49 |
| YY8_May_2021 | 71542878 | 10731431700 | 150;150 | 96.27 | 53.9 |
| YY9_May_2021 | 73300180 | 10995027000 | 150;150 | 96.38 | 54.01 |
| YY10_May_2021 | 71489404 | 10723410600 | 150;150 | 96.15 | 54.08 |
| YY11_May_2021 | 72185532 | 10827829800 | 150;150 | 96.17 | 53.99 |
| WH1_May_2021 | 72754400 | 10913160000 | 150;150 | 96.22 | 50.45 |
| WH2_May_2021 | 58660326 | 8799048900 | 150;150 | 96.16 | 52.27 |
| WH3_May_2021 | 71539372 | 10730905800 | 150;150 | 96.29 | 51.33 |
| WH4_May_2021 | 71553442 | 10733016300 | 150;150 | 96.42 | 50.81 |
| WH5_May_2021 | 71644114 | 10746617100 | 150;150 | 96.21 | 51.8 |
| WH6_May_2021 | 71923584 | 10788537600 | 150;150 | 95.95 | 51.79 |
| WH7_May_2021 | 71297480 | 10694622000 | 150;150 | 96.32 | 51.02 |
| WH8_May_2021 | 71376384 | 10706457600 | 150;150 | 96.6 | 50.97 |
| WH9_May_2021 | 71463152 | 10719472800 | 150;150 | 96.23 | 51.98 |
| WH10_May_2021 | 71384962 | 10707744300 | 150;150 | 96.4 | 51.37 |
| WH11_May_2021 | 71395576 | 10709336400 | 150;150 | 96.44 | 51.34 |
| JJ1_May_2021 | 72833006 | 10924950900 | 150;150 | 96.63 | 51.83 |
| JJ2_May_2021 | 73300292 | 10995043800 | 150;150 | 96.35 | 52.83 |
| JJ3_May_2021 | 71402564 | 10710384600 | 150;150 | 96.28 | 52.73 |
| JJ4_May_2021 | 71303034 | 10695455100 | 150;150 | 96.39 | 51.75 |
| JJ5_May_2021 | 71360374 | 10704056100 | 150;150 | 96.17 | 53.32 |
| JJ6_May_2021 | 71457946 | 10718691900 | 150;150 | 96.36 | 53.15 |
| JJ7_May_2021 | 71458808 | 10718821200 | 150;150 | 96.23 | 51.41 |
| JJ8_May_2021 | 71595736 | 10739360400 | 150;150 | 96.51 | 51.63 |
| JJ9_May_2021 | 71798758 | 10769813700 | 150;150 | 96.33 | 52.87 |
| JJ10_May_2021 | 71632670 | 10744900500 | 150;150 | 96.22 | 52.55 |
| JJ11_May_2021 | 71691830 | 10753774500 | 150;150 | 96.21 | 52.88 |
| NJ1_May_2021 | 71289828 | 10693474200 | 150;150 | 96.31 | 56.94 |
| NJ2_May_2021 | 71368332 | 10705249800 | 150;150 | 96.4 | 54.48 |
| NJ3_May_2021 | 71449390 | 10717408500 | 150;150 | 96.31 | 57.65 |
| NJ4_May_2021 | 71457454 | 10718618100 | 150;150 | 96.43 | 53.19 |
| NJ5_May_2021 | 71578870 | 10736830500 | 150;150 | 96.43 | 53.51 |
| NJ6_May_2021 | 71674538 | 10751180700 | 150;150 | 96.43 | 52.52 |
| NJ7_May_2021 | 73003662 | 10950549300 | 150;150 | 95.97 | 54.45 |
| NJ8_May_2021 | 71387560 | 10708134000 | 150;150 | 96.35 | 54.73 |
| NJ9_May_2021 | 73092238 | 10963835700 | 150;150 | 96.29 | 53.08 |
| NJ12_May_2021 | 71406928 | 10711039200 | 150;150 | 96.26 | 52.35 |
| NJ13_May_2021 | 71534904 | 10730235600 | 150;150 | 96.18 | 53.99 |
| NJ14_May_2021 | 71454004 | 10718100600 | 150;150 | 96.05 | 52.17 |

**Supplementary Table 20: Sample grouping information for co-assembly.**

| **Region** | **January 2020** | **May-June 2020** | **October 2020** | **May 2021** |
| --- | --- | --- | --- | --- |
| Yueyang | Group1 Yueyang Jan. 2020 | Group5 Yueyang May-Jun. 2020 | Group9 Yueyang Oct. 2020 | Group13 Yueyang May 2021 |
| Wuhan | Group2 Wuhan Jan.2020 | Group6 Wuhan May-Jun.2020 | Group10 Wuhan Oct.2020 | Group14 Wuhan May2021 |
| Jiujiang | Group3 Jiujiang Jan. 2020 | Group7 Jiujiang May-Jun. 2020 | Group11 Jiujiang Oct. 2020 | Group15 Jiujiang May 2021 |
| Nanjing | Group4 Nanjing Jan. 2020 | Group8 Nanjing May-Jun. 2020 | Group12 Nanjing Oct. 2020 | Group16 Nanjing May 2021 |

**Supplementary Table 21: MAGs mapping ratio in each sample.**

| **Sample** | **Mapped** | **Reads** | **Ratio** |
| --- | --- | --- | --- |
| JJ1_10_20 | 37800377 | 71114054 | 0.531545804 |
| JJ1_5_21 | 34617179 | 72833006 | 0.475295212 |
| JJ10_10_20 | 38520267 | 71056212 | 0.542109774 |
| JJ10_5_21 | 38518917 | 71632670 | 0.537728344 |
| JJ11_10_20 | 35680380 | 64871800 | 0.550013719 |
| JJ11_5_21 | 39081202 | 71691830 | 0.545127694 |
| JJ2_10_20 | 39241421 | 71115840 | 0.551795788 |
| JJ2_5_21 | 38661463 | 73300292 | 0.527439413 |
| JJ3_10_20 | 39997685 | 71215382 | 0.561643902 |
| JJ3_5_21 | 37523459 | 71402564 | 0.52551977 |
| JJ4_10_20 | 35555358 | 65524764 | 0.54262474 |
| JJ4_5_21 | 37569814 | 71303034 | 0.526903441 |
| JJ5_10_20 | 39212560 | 72336160 | 0.542087941 |
| JJ5_5_21 | 39281651 | 71360374 | 0.550468682 |
| JJ6_10_20 | 35733714 | 64781816 | 0.551600992 |
| JJ6_5_21 | 38222605 | 71457946 | 0.534896497 |
| JJ7_10_20 | 40149262 | 72543334 | 0.55345212 |
| JJ7_5_21 | 38537270 | 71458808 | 0.539293491 |
| JJ8_10_20 | 40552209 | 72667490 | 0.558051599 |
| JJ8_5_21 | 38360295 | 71595736 | 0.535790218 |
| JJ9_10_20 | 37703534 | 67853382 | 0.555661824 |
| JJ9_5_21 | 40294283 | 71798758 | 0.561211421 |
| YY1_10_20 | 39415332 | 71406760 | 0.551983202 |
| YY1_5_21 | 33039259 | 71540854 | 0.46182366 |
| YY10_10_20 | 38332894 | 71890164 | 0.533214725 |
| YY10_5_21 | 31315896 | 71489404 | 0.438049477 |
| YY11_10_20 | 44818281 | 69913042 | 0.641057515 |
| YY11_5_21 | 34371041 | 72185532 | 0.476148614 |
| YY2_10_20 | 36142417 | 69134722 | 0.522782416 |
| YY2_5_21 | 32845448 | 71668130 | 0.458299219 |
| YY3_10_20 | 44793178 | 71584932 | 0.625734729 |
| YY3_5_21 | 33659391 | 72808866 | 0.462297971 |
| YY4_10_20 | 42138213 | 71416524 | 0.5900345 |
| YY4_5_21 | 32957282 | 71336814 | 0.461995429 |
| YY5_10_20 | 39798100 | 69334482 | 0.574001548 |
| YY5_5_21 | 34679800 | 71528622 | 0.484838083 |
| YY6_10_20 | 41341050 | 71744666 | 0.576224719 |
| YY6_5_21 | 31379005 | 71470088 | 0.43905088 |
| YY7_10_20 | 41826325 | 72434948 | 0.57743294 |
| YY7_5_21 | 34092962 | 70762284 | 0.48179567 |
| YY8_10_20 | 42389764 | 73476382 | 0.576916866 |
| YY8_5_21 | 33710537 | 71542878 | 0.471193471 |
| YY9_10_20 | 41333230 | 73066740 | 0.565691449 |
| YY9_5_21 | 34195941 | 73300180 | 0.466519195 |
| NJ1_10_20 | 41079299 | 71222954 | 0.576770503 |

**Supplementary Table 21 continued**

| **Sample** | **Mapped** | **Reads** | **Ratio** |
| --- | --- | --- | --- |
| NJ1_5_21 | 40330873 | 71289828 | 0.565731103 |
| NJ10_10_20 | 38704073 | 71500306 | 0.54131339 |
| NJ12_10_20 | 38665980 | 71563574 | 0.540302529 |
| NJ12_5_21 | 40255353 | 71406928 | 0.563745761 |
| NJ13_10_20 | 38302488 | 71584522 | 0.535066617 |
| NJ13_5_21 | 39558206 | 71534904 | 0.55299167 |
| NJ14_5_21 | 39513110 | 71454004 | 0.552986646 |
| NJ14_10_20 | 37324195 | 70856110 | 0.52676043 |
| NJ2_10_20 | 36314115 | 65974150 | 0.550429449 |
| NJ2_5_21 | 39389774 | 71368332 | 0.551922301 |
| NJ3_10_20 | 38704793 | 71010838 | 0.545054728 |
| NJ3_5_21 | 38581545 | 71449390 | 0.539984246 |
| NJ4_10_20 | 37957418 | 70623582 | 0.537460957 |
| NJ4_5_21 | 38446678 | 71457454 | 0.538035934 |
| NJ5_10_20 | 38186636 | 71716718 | 0.532464913 |
| NJ5_5_21 | 40316279 | 71578870 | 0.563242742 |
| NJ6_10_20 | 34762198 | 64456828 | 0.539309784 |
| NJ6_5_21 | 40125205 | 71674538 | 0.559825094 |
| NJ7_10_20 | 38337762 | 71466836 | 0.536441294 |
| NJ7_5_21 | 39971031 | 73003662 | 0.547520904 |
| NJ8_10_20 | 39889567 | 71542046 | 0.557568161 |
| NJ8_5_21 | 39264968 | 71387560 | 0.550025355 |
| NJ9_10_20 | 38097986 | 71441760 | 0.53327334 |
| NJ9_5_21 | 41108842 | 73092238 | 0.56242418 |
| NJ2_1_20 | 37871650 | 79083688 | 0.478880676 |
| NJ4_1_20 | 41373687 | 79376240 | 0.521235158 |
| NJ10_1_20 | 45217148 | 79428462 | 0.569281425 |
| NJ11_1_20 | 38255709 | 71220502 | 0.537144613 |
| NJ1_1_20 | 37949673 | 79076936 | 0.479908238 |
| NJ3_1_20 | 41646596 | 78767232 | 0.528729967 |
| NJ5_1_20 | 34469278 | 76577864 | 0.450120651 |
| NJ6_1_20 | 38467441 | 78193872 | 0.491949561 |
| NJ7_1_20 | 39633590 | 78170970 | 0.507011618 |
| NJ8_1_20 | 40087397 | 74472512 | 0.538284475 |
| NJ9_1_20 | 41042719 | 79046694 | 0.519221196 |
| NJ12_1_20 | 43325429 | 79510030 | 0.544905203 |
| NJ13_1_20 | 35198086 | 69314096 | 0.507805598 |
| NJ14_1_20 | 40199679 | 79617562 | 0.504909696 |
| JJ1_1_20 | 39784526 | 78139140 | 0.509149781 |
| JJ2_1_20 | 43903153 | 78195306 | 0.561455096 |
| JJ3_1_20 | 45591908 | 78052232 | 0.58412049 |
| JJ4_1_20 | 41569610 | 78101934 | 0.532248151 |
| JJ5_1_20 | 39758140 | 69243474 | 0.574178875 |
| JJ6_1_20 | 44521247 | 79779930 | 0.558050715 |
| JJ7_1_20 | 41796367 | 78075548 | 0.535332355 |
| JJ8_1_20 | 43841445 | 79756010 | 0.549694562 |
| JJ9_1_20 | 43352842 | 79760954 | 0.543534647 |

**Supplementary Table 21 continued**

| **Sample** | **Mapped** | **Reads** | **Ratio** |
| --- | --- | --- | --- |
| JJ10_1_20 | 42518814 | 78075620 | 0.544585032 |
| JJ11_1_20 | 43437894 | 79567924 | 0.545922173 |
| YY1_1_20 | 38227550 | 79133044 | 0.483079483 |
| YY2_1_20 | 37539903 | 78123158 | 0.480522088 |
| YY3_1_20 | 46006210 | 78727318 | 0.584374156 |
| YY4_1_20 | 39194715 | 78438324 | 0.499688328 |
| YY5_1_20 | 48732118 | 78591018 | 0.62007236 |
| YY6_1_20 | 40904892 | 78608586 | 0.520361631 |
| YY7_1_20 | 45727247 | 79268574 | 0.576864761 |
| YY8_1_20 | 44157886 | 79505708 | 0.55540523 |
| YY9_1_20 | 46380463 | 79354750 | 0.584469903 |
| YY10_1_20 | 44161574 | 79338270 | 0.556623859 |
| JJ1_6_20 | 54879372 | 79751744 | 0.688127547 |
| JJ2_6_20 | 39680652 | 68824614 | 0.576547396 |
| WH1_1_20 | 48177055 | 78180824 | 0.616225981 |
| WH2_1_20 | 41088845 | 79039156 | 0.519854299 |
| WH3_1_20 | 41866001 | 78919374 | 0.530490789 |
| WH4_1_20 | 39589336 | 79422832 | 0.498462911 |
| WH6_1_20 | 30176025 | 78193630 | 0.385914108 |
| WH7_1_20 | 35664268 | 78165092 | 0.456268484 |
| JJ3_6_20 | 33753362 | 73210832 | 0.461043278 |
| WH8_1_20 | 47358401 | 73432548 | 0.644923842 |
| WH9_1_20 | 37403749 | 79055272 | 0.473134151 |
| WH10_1_20 | 34513278 | 78673894 | 0.438687807 |
| WH11_1_20 | 42577304 | 78403104 | 0.543056357 |
| H2_1_20 | 39507594 | 79250758 | 0.498513768 |
| WH1_5_20 | 33567266 | 79030862 | 0.424736175 |
| WH2_5_20 | 53366828 | 74968050 | 0.711860959 |
| WH3_5_20 | 45493227 | 65940010 | 0.689918412 |
| WH4_5_20 | 49449142 | 70634490 | 0.700070773 |
| WH5_5_20 | 46113824 | 70413136 | 0.65490371 |
| JJ4_6_20 | 51622661 | 70325502 | 0.734053217 |
| WH6_5_20 | 48019273 | 65637616 | 0.731581613 |
| JJ5_6_20 | 44701389 | 65376776 | 0.683750282 |
| WH7_5_20 | 44989912 | 78600162 | 0.572389558 |
| WH8_5_20 | 48660967 | 79418794 | 0.612713497 |
| WH9_5_20 | 56989801 | 79043404 | 0.720993759 |
| WH10_5_20 | 54184276 | 79190290 | 0.684228786 |
| WH11_5_20 | 52362701 | 79678500 | 0.657174784 |
| H1_5_20 | 53104227 | 79494576 | 0.668023275 |
| H2_5_20 | 49473873 | 79686828 | 0.620853838 |
| H3_5_20 | 50593696 | 73849126 | 0.685095393 |
| NJ1_6_20 | 43707539 | 78957498 | 0.553557801 |
| NJ2_6_20 | 47299238 | 74949746 | 0.631079363 |
| NJ3_6_20 | 45161692 | 78109448 | 0.578184754 |
| NJ4_6_20 | 42192449 | 77370054 | 0.545333069 |
| NJ5_6_20 | 43749022 | 76595922 | 0.571166465 |

**Supplementary Table 21 continued**

| **Sample** | **Mapped** | **Reads** | **Ratio** |
| --- | --- | --- | --- |
| NJ6_6_20 | 50307798 | 79042496 | 0.6364652 |
| NJ7_6_20 | 43619844 | 78087586 | 0.558601517 |
| NJ8_6_20 | 42906130 | 78546932 | 0.546248325 |
| NJ9_6_20 | 43723632 | 79419552 | 0.550539897 |
| NJ10_6_20 | 46304087 | 79381492 | 0.583310868 |
| NJ11_6_20 | 43517232 | 78352530 | 0.555403023 |
| NJ12_6_20 | 34147666 | 64775136 | 0.527172432 |
| JJ7_6_20 | 45308811 | 78505998 | 0.577138208 |
| JJ8_6_20 | 45615650 | 79208150 | 0.575895915 |
| JJ9_6_20 | 45941215 | 72919596 | 0.630025638 |
| JJ10_6_20 | 43262735 | 72596580 | 0.595933514 |
| JJ11_6_20 | 44476132 | 79000904 | 0.56298257 |
| NJ13_6_20 | 46355503 | 79514458 | 0.582982066 |
| NJ14_6_20 | 34833003 | 65153262 | 0.534631758 |
| YY1_6_20 | 42542938 | 78063010 | 0.544982034 |
| YY2_6_20 | 40236157 | 79561908 | 0.505721369 |
| YY3_6_20 | 42953984 | 77495180 | 0.554279427 |
| YY4_6_20 | 43378121 | 79621154 | 0.544806484 |
| YY5_6_20 | 38474092 | 70170380 | 0.548295335 |
| YY6_6_20 | 43082097 | 77436046 | 0.556357139 |
| YY7_6_20 | 42854770 | 74928340 | 0.571943406 |
| YY8_6_20 | 44170514 | 79678776 | 0.554357336 |
| YY9_6_20 | 45055322 | 79784018 | 0.564716131 |
| YY10_6_20 | 45119289 | 79678088 | 0.566269725 |
| YY11_6_20 | 40847285 | 72678862 | 0.562024279 |
| WH1_10_20 | 38504947 | 72255962 | 0.532896469 |
| WH1_5_21 | 40761695 | 72754400 | 0.560264328 |
| WH10_10_20 | 32103874 | 60972968 | 0.526526345 |
| WH10_5_21 | 42677699 | 71384962 | 0.597852794 |
| WH11_10_20 | 36132042 | 72541460 | 0.498088155 |
| WH11_5_21 | 40722061 | 71395576 | 0.57037233 |
| WH2_10_20 | 37796865 | 72486890 | 0.521430358 |
| WH2_5_21 | 34728726 | 58660326 | 0.592030907 |
| WH3_10_20 | 38513018 | 72142810 | 0.533844163 |
| WH3_5_21 | 42544014 | 71539372 | 0.594693702 |
| WH4_10_20 | 37636410 | 70838882 | 0.531295934 |
| WH4_5_21 | 42777315 | 71553442 | 0.597837278 |
| WH5_10_20 | 38336459 | 71722782 | 0.534508812 |
| WH5_5_21 | 41332189 | 71644114 | 0.576909765 |
| WH6_10_20 | 36546901 | 71540760 | 0.510854246 |
| WH6_5_21 | 41498830 | 71923584 | 0.576985012 |
| WH7_10_20 | 37862892 | 72757820 | 0.520396186 |
| WH7_5_21 | 35382854 | 71297480 | 0.496270752 |
| WH8_10_20 | 35991100 | 71984338 | 0.49998515 |
| WH8_5_21 | 40604170 | 71376384 | 0.568874013 |
| WH9_10_20 | 36185828 | 72409804 | 0.499736583 |
| WH9_5_21 | 43948686 | 71463152 | 0.614983873 |

**Supplementary Table 22: General information of MAGs containing ARG(s).**

Please see Supplementary Table 22 in Supplementary Excel file.

**Supplementary Table 23: General information of MAGs containing VFG(s).**

| **Genome** | **Completeness** | **Contamination** |
| --- | --- | --- |
| B11_6_bin.61.fa | 61.89 | 8.852 |
| B11_bin.17.fa | 85.11 | 6.951 |
| B1_6_bin.32.fa | 80.7 | 3.024 |
| B_2020_10_co_bin.107.fa | 88.13 | 5.736 |
| B_2020_10_co_bin.119.fa | 72.36 | 4.383 |
| B_2020_10_co_bin.73.fa | 61.86 | 5.023 |
| B_2020_10_co_bin.81.fa | 71.56 | 3.061 |
| B_2021_6_co_bin.127.fa | 62.93 | 1.724 |
| B_2021_6_co_bin.48.fa | 58.07 | 4.772 |
| B2_bin.49.fa | 54.08 | 5.412 |
| B3_bin.18.fa | 83.01 | 8.358 |
| B5_6_bin.29.fa | 63.02 | 9.482 |
| B6_6_bin.16.fa | 60.78 | 5.344 |
| B7_bin.31.fa | 83.25 | 2.695 |
| D10_6_bin.12.fa | 92.44 | 3.843 |
| D11_bin.23.fa | 64.7 | 2.009 |
| D1_bin.53.fa | 53.68 | 2.037 |
| D_2020_10_co_bin.124.fa | 59.44 | 1.895 |
| D_2021_6_co_bin.143.fa | 59.38 | 6.014 |
| D_2021_6_co_bin.187.fa | 72.13 | 1.487 |
| D5_6_bin.10.fa | 77.57 | 3.147 |
| D5_bin.52.fa | 57.78 | 2.459 |
| D7_bin.29.fa | 66.96 | 6.299 |
| D9_6_bin.55.fa | 55.75 | 5.485 |
| JJ_12_co_bin.111.fa | 54.65 | 6.063 |
| JJ_12_co_bin.21.fa | 53.6 | 1.724 |
| N10_bin.1.fa | 61.37 | 6.034 |
| N12_6_bin.1.fa | 70.17 | 0 |
| N12_bin.39.fa | 98.44 | 3.022 |
| N14B_bin.4.fa | 67.4 | 3.893 |
| N_2020_10_co_bin.106.fa | 85.73 | 4.809 |
| N_2020_10_co_bin.154.fa | 72.49 | 1.96 |
| N_2020_10_co_bin.17.fa | 60.68 | 8.62 |
| N_2020_10_co_bin.76.fa | 63.61 | 3.801 |
| N_2021_6_co_bin.151.fa | 79.62 | 3.243 |
| N2_6_bin.41.fa | 72.58 | 6.266 |
| N2_6_bin.44.fa | 82.28 | 1.826 |
| N2_bin.33.fa | 87.74 | 3.463 |
| N4_6_bin.28.fa | 61.32 | 6.78 |
| N6_6_bin.41.fa | 61.67 | 2.219 |
| N8_6_bin.18.fa | 79.17 | 4.059 |
| N9_6_bin.19.fa | 50.68 | 9.492 |
| N9_bin.43.fa | 53.78 | 0 |
| NJ_12_co_bin.140.fa | 56.16 | 0.886 |
| NJ_6_co_bin.118.fa | 59.48 | 0 |
| NJ_6_co_bin.218.fa | 80.14 | 9.378 |
| NJ_6_co_bin.78.fa | 72 | 5.696 |

**Supplementary Table 23continued**

| **Genome** | **Completeness** | **Contamination** |
| --- | --- | --- |
| Reads_124_METdvbMAADZ-603_bin.34.fa | 61.66 | 2.446 |
| Reads_126_METdvbMAAEB-605_bin.40.fa | 54.96 | 3.605 |
| Reads_127_METdvbMAAEC-606_bin.11.fa | 53.87 | 5.172 |
| Reads_136_METlmsMAAAIAAA-410_bin.53.fa | 85.18 | 6.079 |
| Reads_138_METlmsMAAAKAAA-413_bin.55.fa | 63.03 | 4.108 |
| Reads_232_METlmsMAAEAAAA-413_bin.15.fa | 51.72 | 6.896 |
| Reads_234_METlmsMAAECAAA-415_bin.15.fa | 68.66 | 2.636 |
| Reads_238_METlmsMAAEGAAA-603_bin.4.fa | 79.83 | 4.197 |
| Reads_238_METlmsMAAEGAAA-603_bin.9.fa | 59.92 | 2.761 |
| Reads_252_METlmsMAAEUAAA-620_bin.66.fa | 77.61 | 4.71 |
| Reads_252_METlmsMAAEUAAA-620_bin.70.fa | 52.52 | 1.63 |
| Reads_254_METlmsMAAEWAAA-622_bin.28.fa | 69.95 | 1.023 |
| Reads_255_METlmsMAAEXAAA-624_bin.48.fa | 86.37 | 0.806 |
| Reads_256_METlmsMAAEYAAA-625_bin.11.fa | 53.93 | 9.226 |
| Reads_256_METlmsMAAEYAAA-625_bin.1.fa | 73.07 | 2.627 |
| Reads_256_METlmsMAAEYAAA-625_bin.86.fa | 99.93 | 0.478 |
| Reads_257_METlmsMAAEZAAA-626_bin.38.fa | 90.69 | 2.851 |
| Reads_257_METlmsMAAEZAAA-626_bin.60.fa | 76.95 | 7.799 |
| Reads_259_METlmsMAAFBAAA-628_bin.34.fa | 98.91 | 1.352 |
| Reads_25_METdvbMAAAL-605_bin.43.fa | 59.24 | 5.172 |
| Reads_27_METdvbMAAAN-607_bin.24.fa | 93.36 | 2.57 |
| Reads_35_METdvbMAAAU-617_bin.48.fa | 67.97 | 4.241 |
| Reads_63_METdvbMAABT-645_bin.72.fa | 51.03 | 7.185 |
| Reads_68_METdvbMAABY-651_bin.21.fa | 71.47 | 5.339 |
| WH10_bin.28.fa | 83.35 | 5.562 |
| WH11_bin.23.fa | 72.32 | 5.01 |
| WH_12_co_bin.102.fa | 98.31 | 3.395 |
| WH_2020_10_co_bin.55.fa | 60.69 | 2.941 |
| WH_2021_6_co_bin.103.fa | 68.5 | 3.087 |
| WH_2021_6_co_bin.127.fa | 76.72 | 2.586 |
| WH_2021_6_co_bin.141.fa | 71.13 | 2.927 |
| WH_2021_6_co_bin.86.fa | 59.23 | 1.62 |
| WH3_bin.16.fa | 56.83 | 4.719 |
| WH3_bin.19.fa | 71.88 | 3.6 |
| WH_3_co_bin.151.fa | 54.26 | 9.195 |
| WH_3_co_bin.153.fa | 70.1 | 8.209 |
| WH_3_co_bin.230.fa | 52.58 | 0 |
| WH_3_co_bin.243.fa | 88.77 | 9.008 |
| WH_3_co_bin.269.fa | 69.82 | 2.586 |
| WH_3_co_bin.339.fa | 95.87 | 2.04 |
| WH_3_co_bin.5.fa | 95.39 | 2.507 |
| WH6_6_bin.18.fa | 65.73 | 8.405 |
| WH8_6_bin.19.fa | 88.87 | 4.733 |
| WH8_bin.32.fa | 67.42 | 3.186 |
| YY_12_co_bin.105.fa | 59.15 | 8.808 |
| YY_12_co_bin.130.fa | 89.44 | 2.165 |
| YY_12_co_bin.174.fa | 94.01 | 0.427 |

**Supplementary Table 23 continued**

| **Genome** | **Completeness** | **Contamination** |
| --- | --- | --- |
| YY_12_co_bin.184.fa | 71.62 | 8.883 |
| YY_12_co_bin.33.fa | 93.72 | 5.028 |
| YY_6_co_bin.124.fa | 82.62 | 3.387 |
| YY_6_co_bin.127.fa | 77.42 | 5.532 |
| YY_6_co_bin.228.fa | 61.2 | 0 |
| YY_6_co_bin.48.fa | 55.54 | 3.095 |
| YY_6_co_bin.94.fa | 78.7 | 4.715 |
| B11_6_bin.62.fa | 55.98 | 3.327 |
| B1_6_bin.10.fa | 56.36 | 1.99 |
| B_2021_6_co_bin.216.fa | 56.21 | 8.33 |
| B3_6_bin.12.fa | 57.06 | 4.31 |
| B6_6_bin.32.fa | 50.24 | 7.546 |
| D_2020_10_co_bin.87.fa | 53.58 | 1.453 |
| D8_bin.4.fa | 67.98 | 4.756 |
| JJ_12_co_bin.178.fa | 65.51 | 8.686 |
| JJ_12_co_bin.36.fa | 100 | 0.717 |
| JJ_6_co_bin.14.fa | 63.38 | 5.339 |
| JJ_6_co_bin.62.fa | 87.87 | 2.39 |
| N12_6_bin.16.fa | 70.15 | 5.889 |
| N13_6_bin.17.fa | 78.32 | 7.943 |
| N14_6_bin.29.fa | 51.17 | 3.596 |
| N1_6_bin.29.fa | 56.89 | 3.448 |
| N_2020_10_co_bin.71.fa | 75.03 | 3.693 |
| N2_6_bin.26.fa | 68.11 | 4.647 |
| N4_6_bin.35.fa | 67.33 | 4.572 |
| N5_6_bin.36.fa | 63.15 | 0 |
| N6_6_bin.22.fa | 74.57 | 5.713 |
| N7_6_bin.44.fa | 77.55 | 6.739 |
| N8_6_bin.44.fa | 65.51 | 1.724 |
| N9_6_bin.40.fa | 52.5 | 1.724 |
| NJ_6_co_bin.140.fa | 52.53 | 0 |
| NJ_6_co_bin.173.fa | 96.71 | 2.035 |
| Reads_119_METdvbMAADU-414_bin.43.fa | 70.84 | 9.881 |
| Reads_122_METdvbMAADX-601_bin.39.fa | 53.94 | 5.454 |
| Reads_125_METdvbMAAEA-604_bin.33.fa | 52.33 | 2.163 |
| Reads_126_METdvbMAAEB-605_bin.33.fa | 66.1 | 5.093 |
| Reads_139_METlmsMAAALAAA-414_bin.60.fa | 54.38 | 1.754 |
| Reads_140_METlmsMAAAMAAA-415_bin.42.fa | 50.63 | 2.998 |
| Reads_142_METlmsMAAAOAAA-601_bin.2.fa | 91.42 | 7.097 |
| Reads_143_METlmsMAAAPAAA-602_bin.36.fa | 86.96 | 5.246 |
| Reads_150_METlmsMAAAWAAA-612_bin.14.fa | 95.8 | 1.018 |
| Reads_150_METlmsMAAAWAAA-612_bin.3.fa | 58.1 | 0 |
| Reads_20_METlmsMAAABAAA-403_bin.10.fa | 58.46 | 2.929 |
| Reads_20_METlmsMAAABAAA-403_bin.2.fa | 74.19 | 5.144 |
| Reads_21_METdvbMAAAH-601_bin.13.fa | 99.63 | 1.232 |
| Reads_22_METdvbMAAAI-602_bin.39.fa | 50.35 | 6.791 |
| Reads_235_METlmsMAAEDAAA-416_bin.14.fa | 65.47 | 3.086 |

**Supplementary Table 23 continued**

| **Genome** | **Completeness** | **Contamination** |
| --- | --- | --- |
| Reads_235_METlmsMAAEDAAA-416_bin.65.fa | 56.52 | 4.44 |
| Reads_237_METlmsMAAEFAAA-602_bin.49.fa | 71.81 | 2.826 |
| Reads_238_METlmsMAAEGAAA-603_bin.89.fa | 64.14 | 4.689 |
| Reads_239_METlmsMAAEHAAA-604_bin.47.fa | 57.58 | 3.89 |
| Reads_240_METlmsMAAEIAAA-605_bin.37.fa | 76.54 | 6.676 |
| Reads_240_METlmsMAAEIAAA-605_bin.43.fa | 75.08 | 2.972 |
| Reads_252_METlmsMAAEUAAA-620_bin.31.fa | 62.68 | 3.752 |
| Reads_253_METlmsMAAEVAAA-621_bin.8.fa | 73.76 | 7.875 |
| Reads_254_METlmsMAAEWAAA-622_bin.21.fa | 56.89 | 0.862 |
| Reads_254_METlmsMAAEWAAA-622_bin.38.fa | 58.8 | 5.866 |
| Reads_254_METlmsMAAEWAAA-622_bin.62.fa | 59.56 | 3.967 |
| Reads_255_METlmsMAAEXAAA-624_bin.53.fa | 70.52 | 6.913 |
| Reads_255_METlmsMAAEXAAA-624_bin.66.fa | 92.99 | 3.182 |
| Reads_256_METlmsMAAEYAAA-625_bin.43.fa | 72.34 | 5.492 |
| Reads_257_METlmsMAAEZAAA-626_bin.11.fa | 87.56 | 3.331 |
| Reads_257_METlmsMAAEZAAA-626_bin.1.fa | 80.05 | 5.641 |
| Reads_257_METlmsMAAEZAAA-626_bin.55.fa | 84.05 | 1.444 |
| Reads_257_METlmsMAAEZAAA-626_bin.73.fa | 62.63 | 0 |
| Reads_258_METlmsMAAFAAAA-627_bin.14.fa | 74.69 | 3.217 |
| Reads_258_METlmsMAAFAAAA-627_bin.39.fa | 95.84 | 2.198 |
| Reads_259_METlmsMAAFBAAA-628_bin.25.fa | 64.81 | 3.305 |
| Reads_25_METdvbMAAAL-605_bin.24.fa | 79.47 | 2.071 |
| Reads_26_METdvbMAAAM-606_bin.26.fa | 53.81 | 1.126 |
| Reads_29_METdvbMAAAP-612_bin.22.fa | 93.93 | 6.676 |
| Reads_31_METdvbMAAAR-614_bin.36.fa | 74.32 | 4.801 |
| Reads_33_METlmsMAAACAAA-404_bin.46.fa | 61.79 | 3.025 |
| Reads_34_METdvbMAAAT-616_bin.20.fa | 87.26 | 6.03 |
| Reads_34_METdvbMAAAT-616_bin.42.fa | 85.75 | 6.312 |
| Reads_35_METdvbMAAAU-617_bin.22.fa | 65.63 | 3.568 |
| Reads_35_METdvbMAAAU-617_bin.31.fa | 77.63 | 5.778 |
| Reads_36_METdvbMAAAV-618_bin.1.fa | 77.13 | 7.019 |
| Reads_36_METdvbMAAAV-618_bin.37.fa | 50.78 | 8.256 |
| Reads_38_METdvbMAAAW-619_bin.8.fa | 56.7 | 3.569 |
| Reads_41_METdvbMAAAZ-622_bin.21.fa | 54.46 | 6.896 |
| Reads_43_METdvbMAABB-625_bin.17.fa | 71.38 | 7.658 |
| Reads_44_METdvbMAABC-626_bin.13.fa | 69.29 | 3.98 |
| Reads_44_METdvbMAABC-626_bin.19.fa | 72.41 | 1.724 |
| WH_12_co_bin.145.fa | 60.63 | 2.002 |
| WH_12_co_bin.207.fa | 56.14 | 4.385 |
| WH_12_co_bin.209.fa | 63.14 | 7.151 |
| WH1_6_bin.41.fa | 77.7 | 4.481 |
| WH_2020_10_co_bin.6.fa | 78.04 | 6.896 |
| WH_3_co_bin.144.fa | 53.2 | 6.133 |
| WH_3_co_bin.190.fa | 50.86 | 6.896 |
| WH_3_co_bin.268.fa | 62.06 | 6.034 |

**Supplementary Table 23 continued**

| **Genome** | **Completeness** | **Contamination** |
| --- | --- | --- |
| WH_3_co_bin.95.fa | 50.86 | 3.988 |
| WH8_bin.35.fa | 81.3 | 5.285 |
| YY_12_co_bin.235.fa | 60.49 | 6.11 |
| YY_6_co_bin.250.fa | 54.14 | 8.733 |

**Supplementary Table 24: General information of MAGs containing both ARG(s) and VFG(s).**

| **Genome** | **Completeness** | **Contamination** |
| --- | --- | --- |
| B11_6_bin.62.fa | 55.98 | 3.327 |
| B1_6_bin.10.fa | 56.36 | 1.99 |
| B_2021_6_co_bin.216.fa | 56.21 | 8.33 |
| B3_6_bin.12.fa | 57.06 | 4.31 |
| B6_6_bin.32.fa | 50.24 | 7.546 |
| D_2020_10_co_bin.87.fa | 53.58 | 1.453 |
| D8_bin.4.fa | 67.98 | 4.756 |
| JJ_12_co_bin.178.fa | 65.51 | 8.686 |
| JJ_12_co_bin.36.fa | 100 | 0.717 |
| JJ_6_co_bin.14.fa | 63.38 | 5.339 |
| JJ_6_co_bin.62.fa | 87.87 | 2.39 |
| N12_6_bin.16.fa | 70.15 | 5.889 |
| N13_6_bin.17.fa | 78.32 | 7.943 |
| N14_6_bin.29.fa | 51.17 | 3.596 |
| N1_6_bin.29.fa | 56.89 | 3.448 |
| N_2020_10_co_bin.71.fa | 75.03 | 3.693 |
| N2_6_bin.26.fa | 68.11 | 4.647 |
| N4_6_bin.35.fa | 67.33 | 4.572 |
| N5_6_bin.36.fa | 63.15 | 0 |
| N6_6_bin.22.fa | 74.57 | 5.713 |
| N7_6_bin.44.fa | 77.55 | 6.739 |
| N8_6_bin.44.fa | 65.51 | 1.724 |
| N9_6_bin.40.fa | 52.5 | 1.724 |
| NJ_6_co_bin.140.fa | 52.53 | 0 |
| NJ_6_co_bin.173.fa | 96.71 | 2.035 |
| Reads_119_METdvbMAADU-414_bin.43.fa | 70.84 | 9.881 |
| Reads_122_METdvbMAADX-601_bin.39.fa | 53.94 | 5.454 |
| Reads_125_METdvbMAAEA-604_bin.33.fa | 52.33 | 2.163 |
| Reads_126_METdvbMAAEB-605_bin.33.fa | 66.1 | 5.093 |
| Reads_139_METlmsMAAALAAA-414_bin.60.fa | 54.38 | 1.754 |
| Reads_140_METlmsMAAAMAAA-415_bin.42.fa | 50.63 | 2.998 |
| Reads_142_METlmsMAAAOAAA-601_bin.2.fa | 91.42 | 7.097 |
| Reads_143_METlmsMAAAPAAA-602_bin.36.fa | 86.96 | 5.246 |
| Reads_150_METlmsMAAAWAAA-612_bin.14.fa | 95.8 | 1.018 |
| Reads_150_METlmsMAAAWAAA-612_bin.3.fa | 58.1 | 0 |
| Reads_20_METlmsMAAABAAA-403_bin.10.fa | 58.46 | 2.929 |
| Reads_20_METlmsMAAABAAA-403_bin.2.fa | 74.19 | 5.144 |
| Reads_21_METdvbMAAAH-601_bin.13.fa | 99.63 | 1.232 |
| Reads_22_METdvbMAAAI-602_bin.39.fa | 50.35 | 6.791 |
| Reads_235_METlmsMAAEDAAA-416_bin.14.fa | 65.47 | 3.086 |
| Reads_235_METlmsMAAEDAAA-416_bin.65.fa | 56.52 | 4.44 |
| Reads_237_METlmsMAAEFAAA-602_bin.49.fa | 71.81 | 2.826 |
| Reads_238_METlmsMAAEGAAA-603_bin.89.fa | 64.14 | 4.689 |
| Reads_239_METlmsMAAEHAAA-604_bin.47.fa | 57.58 | 3.89 |

**Supplementary Table 24 continued.**

| **Genome** | **Completeness** | **Contamination** |
| --- | --- | --- |
| Reads_240_METlmsMAAEIAAA-605_bin.37.fa | 76.54 | 6.676 |
| Reads_240_METlmsMAAEIAAA-605_bin.43.fa | 75.08 | 2.972 |
| Reads_252_METlmsMAAEUAAA-620_bin.31.fa | 62.68 | 3.752 |
| Reads_253_METlmsMAAEVAAA-621_bin.8.fa | 73.76 | 7.875 |
| Reads_254_METlmsMAAEWAAA-622_bin.21.fa | 56.89 | 0.862 |
| Reads_254_METlmsMAAEWAAA-622_bin.38.fa | 58.8 | 5.866 |
| Reads_254_METlmsMAAEWAAA-622_bin.62.fa | 59.56 | 3.967 |
| Reads_255_METlmsMAAEXAAA-624_bin.53.fa | 70.52 | 6.913 |
| Reads_255_METlmsMAAEXAAA-624_bin.66.fa | 92.99 | 3.182 |
| Reads_256_METlmsMAAEYAAA-625_bin.43.fa | 72.34 | 5.492 |
| Reads_257_METlmsMAAEZAAA-626_bin.11.fa | 87.56 | 3.331 |
| Reads_257_METlmsMAAEZAAA-626_bin.1.fa | 80.05 | 5.641 |
| Reads_257_METlmsMAAEZAAA-626_bin.55.fa | 84.05 | 1.444 |
| Reads_257_METlmsMAAEZAAA-626_bin.73.fa | 62.63 | 0 |
| Reads_258_METlmsMAAFAAAA-627_bin.14.fa | 74.69 | 3.217 |
| Reads_258_METlmsMAAFAAAA-627_bin.39.fa | 95.84 | 2.198 |
| Reads_259_METlmsMAAFBAAA-628_bin.25.fa | 64.81 | 3.305 |
| Reads_25_METdvbMAAAL-605_bin.24.fa | 79.47 | 2.071 |
| Reads_26_METdvbMAAAM-606_bin.26.fa | 53.81 | 1.126 |
| Reads_29_METdvbMAAAP-612_bin.22.fa | 93.93 | 6.676 |
| Reads_31_METdvbMAAAR-614_bin.36.fa | 74.32 | 4.801 |
| Reads_33_METlmsMAAACAAA-404_bin.46.fa | 61.79 | 3.025 |
| Reads_34_METdvbMAAAT-616_bin.20.fa | 87.26 | 6.03 |
| Reads_34_METdvbMAAAT-616_bin.42.fa | 85.75 | 6.312 |
| Reads_35_METdvbMAAAU-617_bin.22.fa | 65.63 | 3.568 |
| Reads_35_METdvbMAAAU-617_bin.31.fa | 77.63 | 5.778 |
| Reads_36_METdvbMAAAV-618_bin.1.fa | 77.13 | 7.019 |
| Reads_36_METdvbMAAAV-618_bin.37.fa | 50.78 | 8.256 |
| Reads_38_METdvbMAAAW-619_bin.8.fa | 56.7 | 3.569 |
| Reads_41_METdvbMAAAZ-622_bin.21.fa | 54.46 | 6.896 |
| Reads_43_METdvbMAABB-625_bin.17.fa | 71.38 | 7.658 |
| Reads_44_METdvbMAABC-626_bin.13.fa | 69.29 | 3.98 |
| Reads_44_METdvbMAABC-626_bin.19.fa | 72.41 | 1.724 |
| WH_12_co_bin.145.fa | 60.63 | 2.002 |
| WH_12_co_bin.207.fa | 56.14 | 4.385 |
| WH_12_co_bin.209.fa | 63.14 | 7.151 |
| WH1_6_bin.41.fa | 77.7 | 4.481 |
| WH_2020_10_co_bin.6.fa | 78.04 | 6.896 |
| WH_3_co_bin.144.fa | 53.2 | 6.133 |
| WH_3_co_bin.190.fa | 50.86 | 6.896 |
| WH_3_co_bin.268.fa | 62.06 | 6.034 |
| WH_3_co_bin.95.fa | 50.86 | 3.988 |
| WH8_bin.35.fa | 81.3 | 5.285 |
| YY_12_co_bin.235.fa | 60.49 | 6.11 |
| YY_6_co_bin.250.fa | 54.14 | 8.733 |

**REFERENCES AND NOTES**

1. Hubbell S. The unified neutral theory of biodiversity and biogeography. Princeton University Press, Princeton, 2001.

2. Chen W, Ren K, Isabwe A, Chen H, Liu M, Yang J. Stochastic processes shape microeukaryotic community assembly in a subtropical river across wet and dry seasons. Microbiome. 2019; 7:138.

3. Sloan WT, Lunn M, Woodcock S, Head IM, Nee S, Curtis TP. Quantifying the roles of immigration and chance in shaping prokaryote community structure. Environ. Microbio. 2006; l8:732-740.
